# Supplementary material for: Bayesian Decision Curve Analysis With Bayesdca
Source: Stat Med. 2024 Dec 1;43(30):6042–58. doi: 10.1002/sim.10277 (PMC11639651; doi:10.1002/sim.10277)
Supplement: Supplementary file 1 — Appendix S1. Supplementary methods. [file SIM-43-6042-s001.zip › sim10277-sup-0001-supinfo.pdf]

SUPPLEMENT

Supplementary methods

1 | Informative priors in Bayesian DCA for binary outcomes

In this section, we briefly describe how informative priors can be easily employed within bayesDCA for binary outcomes. These can be advantageous under small effective sample sizes, for instance, for the estimation of EVPI [17]. Additionally, we demonstrate the visual prior checks available within bayesDCA to aid in understanding the implications of the chosen prior parameters [47].

In the proposed Bayesian DCA approach, each parameter of interest (sensitivity, specificity, and prevalence) follows an independent Beta distribution – a priori and a posteriori. For interpretability, we parameterize the Beta priors using the prior mean and prior sample size (or "prior strength"). For instance, suppose we state a priori that the prevalence p follows a Beta distribution with mean μ and sample size (or strength) η. Then $p \sim \text{Beta}(\mu\,\eta,\ (1-\mu)\,\eta)$ a priori.

In DCA for binary outcomes, sensitivity (Se) decreases as the decision threshold increases, and the reverse is true for specificity. This prior knowledge motivates a threshold-varying prior implemented within bayesDCA as follows: for a given decision strategy (e.g., a diagnostic model), let Se_t be the (prior) sensitivity at the decision threshold t. Define an "ignorance region" $R = (a, b)$ with $0 \leq t_{\text{min}} < a < b < t_{\text{max}} < 1$ where we have no prior knowledge about the sensitivity of the decision strategy. Here t_{min} is the lowest decision threshold considered in the DCA (usually 0) and t_{max} the highest. At t_{min}, we strongly believe the sensitivity is high, which translates into a high prior mean and a large prior sample size. As the decision threshold increases, the sensitivity should decrease, though by an uncertain amount. Hence, we progressively decrease both the prior mean and prior sample size. As the decision threshold approaches a, the prior mean approaches 0.5 and the prior sample size approaches 2, which together represent the vague $\text{Beta}(1, 1)$ prior – fixed for any t between a and b. The prior mean starts decreasing again for $t > b$, reaching its minimum at t_{max}, whereas the prior sample size now increases (at the same rate it previously decreased for $t < a$). Since we expect an opposite behaviour for the specificity, we set its prior mean to be the opposite of the prior mean sensitivity, for any given threshold. Supplementary Figure (S1) shows the resulting prior.

When using this threshold-varying prior, the default ignorance region R within bayesDCA is $(0.25,\ 0.75) \times t_{\text{max}}$. Optionally, the user may specify prior mean and sample size other than 0.5 and 2, respectively, to be fixed within this region. The default prior mean sensitivities are 0.99 and 0.01 at t_{min} and t_{max}, respectively. The default prior sample size for sensitivity and specificity is 5 at both t_{min} and t_{max} and 2 within the ignorance region. We employ linear interpolation to get prior means and sample sizes at decision thresholds between t_{min} an a and between b and t_{max}. We remain vague about the prevalence by default as it is highly context-dependent, but users should set more reasonable parameters for their problem.

The threshold-varying prior above is not particularly informative, in the sense that it can hardly impact the net benefit estimates for reasonably large datasets (e.g., with 100 events or more). Yet, it is a starting point from which we can build more informative priors that make sense in any given context. For instance, suppose we wish to set the prior sensitivity to linearly decrease with the decision threshold, without any ignorance region; we also believe that the prevalence is likely between 20% and 40%, with the best guess at 30%. With bayesDCA, it is easy to employ such priors and visualize its implications, shown in Supplementary Figure (S2).

Notice that, because we "turned off" the ignorance region, the default prior sample size for sensitivity and specificity is fixed at 5 (default). Still, there is considerable prior uncertainty for these parameters depending on the decision threshold. The prior on the prevalence parameter completely determines the prior net benefit for the Treat all strategy (recall that $NB_{\text{all}} = p - w_t\,(1-p)$ where $w_t = t/(1-t)$). However, the prior net benefit of a hypothetical model-based strategy (to be assessed using DCA) closely follows the Treat all strategy for many thresholds. Even though our prior sample size for sensitivity and specificity is not large, the prior prevalence has a particularly strong effect on the implied prior net benefit for all decision strategies. In particular, it sets an upper bound on the prior net benefit – here, around 0.4. In this example, the effect of the prior sensitivity and specificity is more pronounced at higher thresholds, where the prior net benefit from the Treat all and model-based strategies start to diverge more notably.

Though the user of bayesDCA may employ arbitrary prior parameters if desired, the above examples illustrate what is easily accessible in terms of threshold-varying priors within the package. The ignorance region enables prior vagueness for an arbitrary portion of the decision curve while still taking advantage of prior knowledge about sensitivity and specificity at very high and very low thresholds. The prior prevalence has en effect of restricting the range of prior net benefit that is plausible for any given sensitivity and specificity. Future work may further develop prior distributions based on prior knowledge about discrimination

and calibration as well as on ideas from signal detection theory[48]. Finally, the implications of any prior choices can be visualized with prior checks.

2 | Informative priors preserve EVPI monotonic behaviour

To show how we can leverage informative priors to preserve the expected monotonic behaviour of the EVPI, we adapt the simulation code published in Sadatsafavi et al. (2023)[17]. Briefly, using an example model trained with a subset of the GUSTO-I trial data, we run DCA and EVPI calculations in an external dataset of increasing sample size (held out GUSTO-I data) as previously described[17]. We repeat this procedure for four decision thresholds: 0.01, 0.02, 0.05, and 0.1. Previously, Sadatsafavi et al. (2023)[17] identified unexpected behaviour of EVPI for the 0.01 threshold: the EVPI would increase and then decrease with sample size (see Figure 5 in the publication).

Here, we repeat their simulation but adding two methods: bayesDCA with uniform $\text{Beta}(1, 1)$ priors on all parameters ("BayesDCA (uniform)") and bayesDCA with informative priors ("BayesDCA (informative)"). For the informative case, we employed bayesDCA's framework for threshold-varying priors described in the previous section (using the .get_prior_parameters() function implemented in bayesDCA). A summary of the resulting prior distributions for sensitivity and specificity is shown in Supplementary Table (S3). The prior sample size was fixed at 10 and prevalence used a uniform Beta(1, 1) prior. Supplementary Figure (S3) shows the results.

For all methods except bayesDCA with informative priors, the non-monotonic behaviour is observed at the lowest decision threshold of 0.01. The informative prior recovers the expected monotonic behaviour, adequately indicating higher EVPI for lower sample sizes in all decision thresholds. Thus, bayesDCA offers the ability to leverage prior information to improve EVPI calculations in settings with small effective sample sizes.

3 | Implementation Decision Rules

Given an observed DCA result and an established Standard of Core (SoC), a decision maker (DM) must decide whether to implement a new clinical prediction model. The SoC may be another prediction model, binary test, or treat all/none. Suppose the DM is willing to implement the new model if its P(best) is greater than γ for $\gamma \in (0, 1)$. A risk-neutral DM takes $\gamma = 0.5$, whereas a risk-averse DM takes $\gamma > 0.5$. Varying risk profiles give rise to different implementation decision rules $\phi(\gamma) = \mathbb{1}\{\text{P(best)} > \gamma\}$. Given an observed dataset, P(best) is estimated as a posterior probability and $\phi(\gamma)$ outputs 1 if such a probability exceeds γ and 0 otherwise. Provided the posterior distributions are symmetric around their mean, setting $\gamma = 0.5$ is analogous to choosing the clinical decision strategy with the highest observed net benefit.

Now, any rational DM implements the new model when the expected utility of implementing, $\mathbb{E}[U_1]$, is higher than the expected utility of not implementing, $\mathbb{E}[U_0]$. Since any net benefit gain fully justifies preferring one clinical decision strategy over another, these utilities depend on whether $\delta_{NB} > 0$ where $\delta_{NB} = \text{NB}_{\text{new model}} - \text{NB}_{\text{SoC}}$. Thus, requiring $\mathbb{E}[U_1] > \mathbb{E}[U_0]$ is equivalent to:

$$\mathbb{E}\Big[U_1 \mid \delta_{NB} > 0\Big]\text{P(best)} + \mathbb{E}\Big[U_1 \mid \delta_{NB} < 0\Big](1 - \text{P(best)}) \; > \; \mathbb{E}\Big[U_0 \mid \delta_{NB} > 0\Big]\text{P(best)} + \mathbb{E}\Big[U_0 \mid \delta_{NB} < 0\Big](1 - \text{P(best)})$$

Rearranging terms we get

$$\frac{\text{P(best)}}{1 - \text{P(best)}} > \frac{\mathbb{E}\Big[U_0 \mid \delta_{NB} < 0\Big] - \mathbb{E}\Big[U_1 \mid \delta_{NB} < 0\Big]}{\mathbb{E}\Big[U_1 \mid \delta_{NB} > 0\Big] - \mathbb{E}\Big[U_0 \mid \delta_{NB} > 0\Big]} \tag{21}$$

which defines a rational implementation decision threshold. Recall, however, that our example DM is willing to implement when $\text{P(best)} > \gamma \iff \frac{\text{P(best)}}{1 - \text{P(best)}} > w(\gamma)$ where $w(\gamma) = \gamma/(1 - \gamma)$. If our example DM is rational, then their implementation decision threshold is described by (21) so that their choice of γ implies

$$\mathbb{E}\Big[U_0 \mid \delta_{NB} < 0\Big] - \mathbb{E}\Big[U_1 \mid \delta_{NB} < 0\Big] = w(\gamma) \times \left\{\mathbb{E}\Big[U_1 \mid \delta_{NB} > 0\Big] - \mathbb{E}\Big[U_0 \mid \delta_{NB} > 0\Big]\right\} \tag{22}$$

Notice that the left-hand side of the expression above, $\mathbb{E}[U_0 \mid \delta_{NB} < 0] - \mathbb{E}[U_1 \mid \delta_{NB} < 0]$, can be seen as the utility loss due to implementing when the new model is inferior, which we refer to as the harm of an inadequate implementation. Conversely, the expectation on the right-hand side $\mathbb{E}[U_1 \mid \delta_{NB} > 0] - \mathbb{E}[U_0 \mid \delta_{NB} > 0]$ represents the harm from missing an adequate implementation – i.e., failing to implement a superior new model. As risk-neutral DMs take $\gamma = 0.5 \iff w(\gamma) = 1$, they consider these harms to be exactly equal. A risk-averse DM takes $\gamma > 0.5 \iff w(\gamma) > 1$ so that implementing a new model that is inferior to the current SoC is seen as worse than failing to implement a superior new model.

Further, equation (22) also enables putting harms on the same scale, which is helpful to compare implementation decision rules across a range of risk preferences. Let $\mathcal{H}$ denote the space of possible states of nature that generate the data used for DCA – i.e., the underlying distributions of predictions and outcome. Suppose there are two fundamental states in $\mathcal{H}$, h_1 and h_2. Under h_1, the new model is superior to the SoC and the correct decision is to implement. Thus, we can calculate the Frequentist Risk of any decision rule ϕ as:

$$R_{h_1}(\phi) = \left[1 - \mathbb{E}_{h_1}\left[\phi\right]\right] \times \left[\mathbb{E}[U_1 \mid \delta_{NB} > 0] - \mathbb{E}[U_0 \mid \delta_{NB} > 0]\right] \tag{23}$$

Conversely, under h_2, the new model is inferior to the SoC so the correct decision is not to implement, in which case the Frequentist Risk is:

$$R_{h_2}(\phi) = \mathbb{E}_{h_2}\left[\phi\right] \times \left[\mathbb{E}[U_0 \mid \delta_{NB} < 0] - \mathbb{E}[U_1 \mid \delta_{NB} < 0]\right] \tag{24}$$

$$= \mathbb{E}_{h_2}\left[\phi\right] \times w(\gamma) \times \left[\mathbb{E}[U_1 \mid \delta_{NB} > 0] - \mathbb{E}[U_0 \mid \delta_{NB} > 0]\right] \tag{25}$$

Trivially, if $h = h_1$, the Frequentist Risk is minimized by an implementation decision rule that always implements. If $h = h_2$, a decision rule that never implements is deemed optimal. In practice, however, we do not know h. The Bayesian solution to compare decision rules is to place a prior π_0 over $\mathcal{H}$ and average $h \in \mathcal{H}$ out according to that prior[49]. The Bayes Risk is then defined as

$$r_{\pi_0}(\phi) = \mathbb{E}_{h \sim \pi_0}\left[R_h(\phi)\right] = \pi_0(h_1) \cdot R_{h_1}(\phi) + [1 - \pi_0(h_1)] \cdot R_{h_2}(\phi) \tag{26}$$

The Bayes rule $\phi^* = \arg\min_\phi r_{\pi_0}(\phi)$ is then the optimal implementation decision rule. By the definition (26) and expanding (23) and (25), the Bayes rule satisfies:

$$\phi^* = \arg\min_\phi \left\{\pi_0(h_1) \times (1 - \mathbb{E}_{h_1}\left[\phi\right]) + [1 - \pi_0(h_1)] \times \mathbb{E}_{h_2}\left[\phi\right] \times w(\gamma)\right\} \tag{27}$$

We can see that the expected utility terms cancel out, as accounting for the risk aversion term $w(\gamma)$ allows putting Frequentist Risks on the same utility scale. Putting a uniform prior over $\mathcal{H}$ sets $\pi_0(h_1) = 0.5$ so that the Bayes rule further simplifies to:

$$\phi^* = \arg\min_\phi \left\{(1 - \mathbb{E}_{h_1}\left[\phi\right]) + \mathbb{E}_{h_2}\left[\phi\right] \times w(\gamma)\right\} \tag{28}$$

The best implementation decision strategy depends on risk aversion

Based on the previous section, we can now evaluate the best implementation decision strategy using the following simulation. Suppose that we have a binary outcome with a prevalence of 19.5%, generated according to the data-generating process below.

$$\begin{aligned} &X_1, X_2, X_3, X_4 \overset{iid}{\sim} \mathcal{N}(0,1) \\ &\eta = -3 + 0.1 \times X_1 + 1.4 \times X_2 + -1.75 \times X_3 + 2 \times X_4 \\ &Y \sim \text{Bernoulli}(1/[1 + \exp(-\eta)]) \end{aligned} \tag{29}$$

We then need to evaluate two competing prediction models, model A and model B, whose linear predictors are generated as follows:

$$\begin{aligned} \widehat{\eta}_A &= -1.6 + 0.12 \times X_1 + 1.55 \times X_2 \\ \widehat{\eta}_B &= -1.7 + 0.075 \times X_1 + -1.1 \times X_3 \end{aligned}$$

The average predicted probability from model A is 24%, with a calibration slope of 0.5 and an AUC of 0.7. The average prediction from model B is 20%, with a calibration slope of 0.92 and AUC of 0.75. Both models A and B are clinically useful for clinical decision thresholds between 10% and 30% – Figure S12. However, notice that the difference in net benefit is numerically tiny (< 0.025). If we were to naively perform traditional null hypothesis significance testing (NHST) on the net benefit scale, a substantial sample size would be required to achieve a desirable statistical power.

Now, suppose model A is the SoC and we collect data to decide whether to implement model B. This scenario corresponds to h_1, where the correct decision is to implement. Across 1000 simulation runs, we generate data according to (29), perform Bayesian DCA, and apply different implementation decision rules. The baseline implementation rule implements if the estimated net benefit from model B is the highest among all competing strategies. Other implementation rules are based on different empirical cutoffs for P(best). We then repeat this process by inverting the roles: model B is treated as the SoC and model A is treated as the new model. This second scenario corresponds to h_2, where the correct decision is not to implement. Varying sample size and risk aversion parameter γ, we can then compute the Bayes Risk as in (28) for each implementation decision rule.

The results are shown in Figure (S13). When the DM is risk neutral ($\gamma = 0.5$), the baseline implementation decision rule based on point estimates minimizes the Bayes risk, regardless of sample size. Up to numerical differences, P(best) $> 50\%$ is equivalent to the baseline rule. Under risk aversion ($\gamma = 0.8$ or $\gamma = 0.9$), implementing based on point estimates alone leads to excess Bayes risk in all scenarios, especially when the sample size is small. This is intuitive: with smaller samples, you expect the point estimate of δ_{NB} to have the wrong sign more often, leading to inappropriate implementations. If the DM is risk averse, these inappropriate implementations are upweighted, leading to excess risk. Importantly, these results clearly show that mistaking risk-averse implementation decision rules as NHST can be highly misleading: the benefit from Bayesian DCA for risk-averse DMs is particularly prominent under smaller sample sizes, when traditional NHST would suffer from very low statistical power.

In summary, here we have shown that the implementation decision rule that minimizes the Bayes risk depends on the DM's risk preferences. As varying risk preferences lead to different Bayes rules, deciding based on point estimates alone is acceptable only if the DM is risk-neutral. Neglecting risk aversion leads to excess Bayes risk, especially for smaller sample sizes.

Supplementary figures

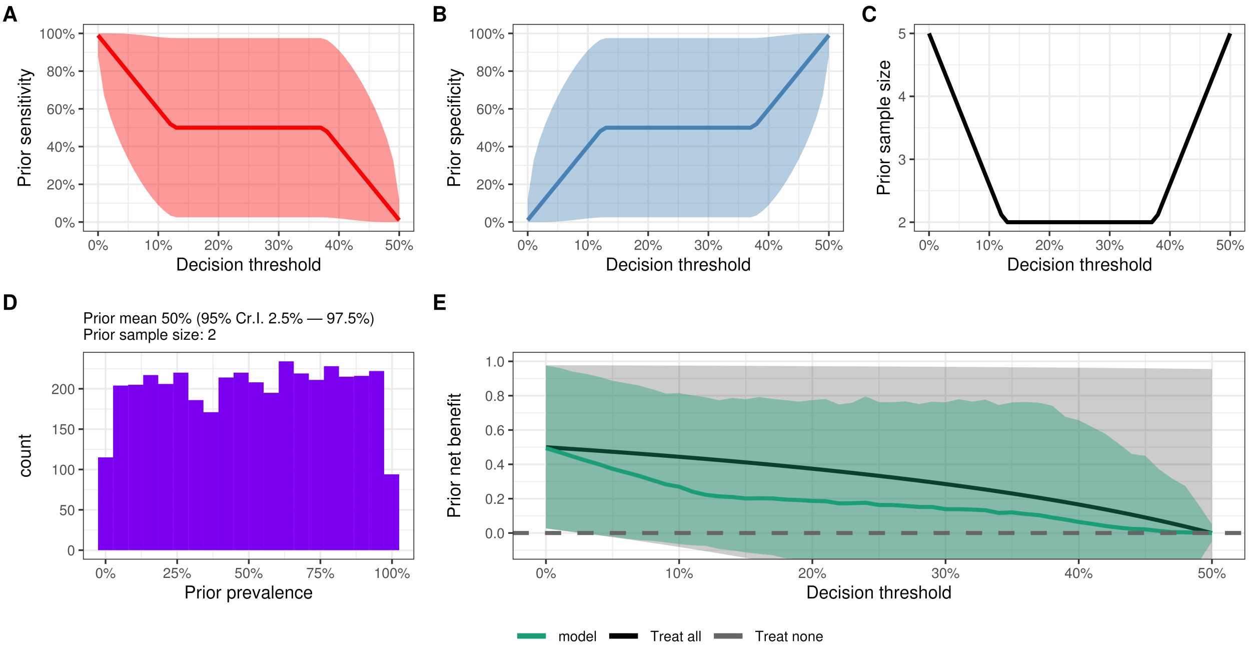

Supplementary Figure S1 **bayesDCA allows easy implementation and visualization of threshold-varying priors. (A)** The prior mean and prior "strength" for sensitivity start high at the lowest decision threshold and decrease linearly until reaching a region of ignorance, where the prior sensitivity is Beta(1, 1) (i.e., uniform). After the region of ignorance, the prior mean starts decreasing again, but the prior strength increases. **(B)** The prior specificity shows the exact opposite behaviour as the prior sensitivity. **(C)** The prior sample size for both sensitivity and specificity defines the prior strength, is high for extreme decision thresholds (i.e., near the minimum and maximum thresholds), and low within the region of ignorance. **(D)** The default prior prevalence is the vague Beta(1,1) as it is highly context-dependent. **(E)** The implications of these priors on the prior net benefit for the Treat all strategy and a hypothetical model-based strategy. By default, the same threshold-varying prior is applied to all decision strategies being analyzed.

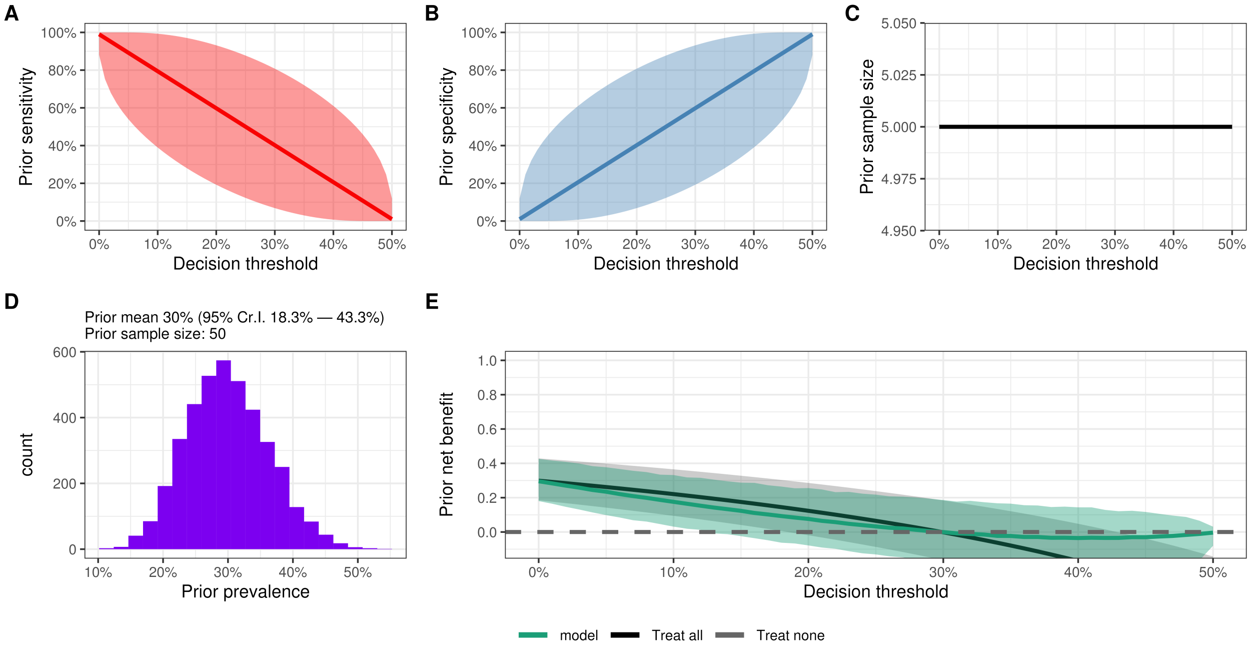

Supplementary Figure S2 **bayesDCA allows easy implementation and visualization of strongly informative threshold-varying priors. (A)** The prior mean sensitivity decreases linearly with the decision threshold. Although prior "strength" is fixed, the prior uncertainty varies and is highest when the prior mean is 50%. **(B)** The prior specificity shows the exact opposite behaviour as the prior sensitivity. **(C)** The prior sample size for both sensitivity and specificity is fixed in this case. **(D)** A prior prevalence suggesting the true prevalence is likely between 20% and 40%, with best guess at 30%. **(E)** The implications of these priors on the prior net benefit for the Treat all strategy and a hypothetical model-based strategy. The prior prevalence restricts the prior net benefit to be mostly below 0.4, and the priors on sensitivity and specificity drive any differences between the Treat all and model-based decision strategies.

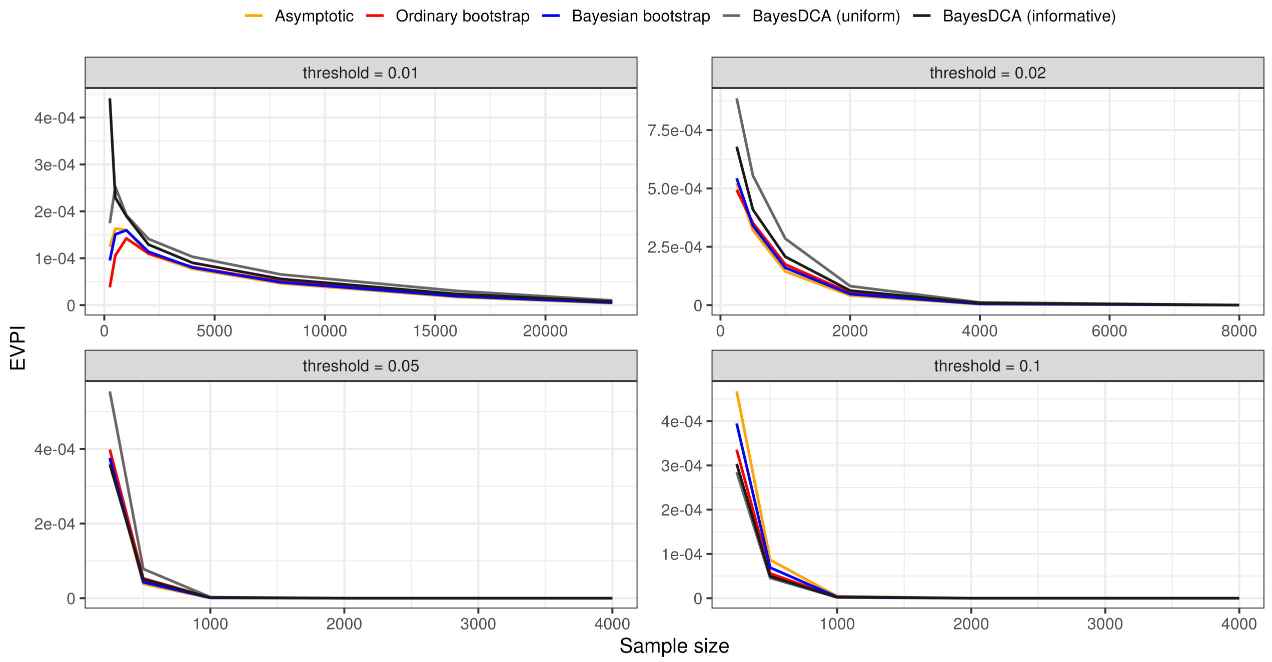

Supplementary Figure S3 **Informative priors preserve EVPI monotonic behaviour.** EVPI simulation adapted from Sadatsafavi et al. (2023)[17] using GUSTO-I trial data as an example (see Figure 5 in the cited manuscript). Each panel is one decision threshold, and the validation sample size is shown on the x-axis. Informative prior parameters for bayesDCA are reported in Table (S3).

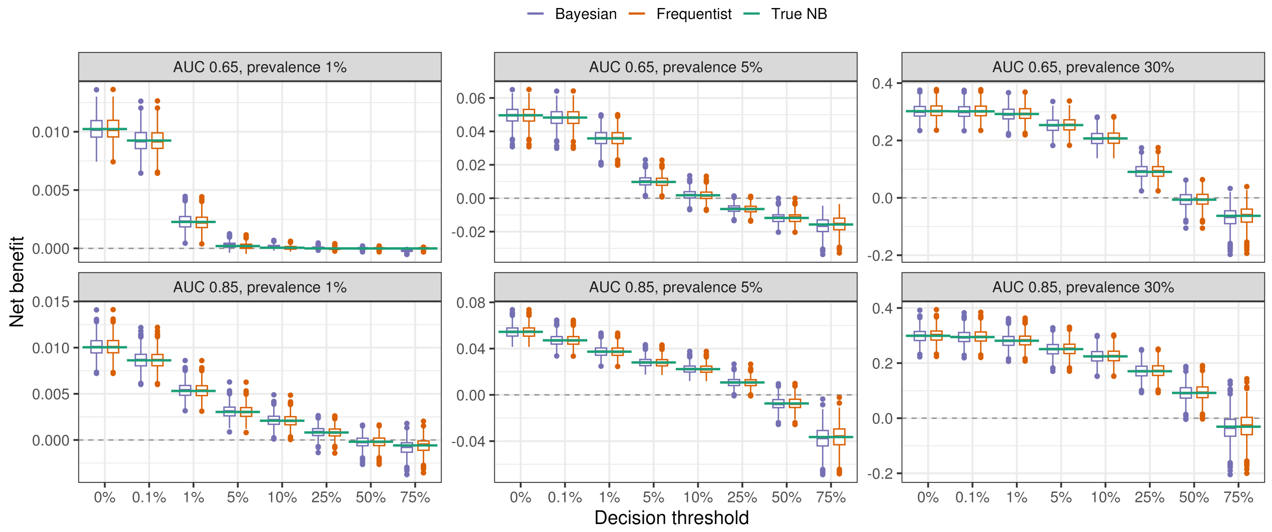

Supplementary Figure S4 **Bayesian and Frequentist DCA for binary outcomes show similar point estimate distributions.** Bayesian DCA was computed using the bayesDCA R package, while the Frequentist alternative used the bootstrap-based rmda package. For each simulation run, DCA was performed for a fixed example model using a simulated test dataset of sample size corresponding to 100 expected events. A total of 1000 Monte Carlo repetitions was run for each setting. The setting AUC corresponds to its maximum achievable AUC. The example model for each setting was fixed to approximate the maximum discrimination of that setting but was miscalibrated (overly extreme risk predictions).

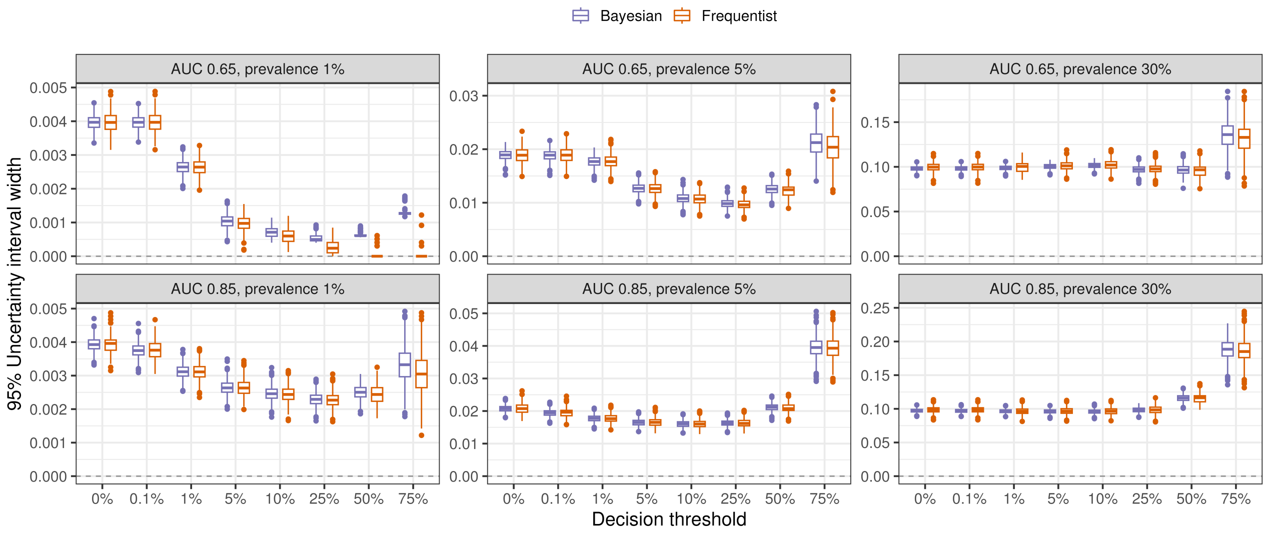

Supplementary Figure S5 **Bayesian and Frequentist DCA for binary outcomes show similar width of uncertainty intervals, except when bootstrap fails.** Bayesian DCA was computed using the bayesDCA R package, while the Frequentist alternative used the bootstrap-based rmda package. For each simulation run, DCA was performed for a fixed example model using a simulated test dataset of sample size corresponding to 100 expected events. A total of 1000 Monte Carlo repetitions was run for each setting. The setting AUC corresponds to its maximum achievable AUC. The example model for each setting was fixed to approximate the maximum discrimination of that setting but was miscalibrated (overly extreme risk predictions).

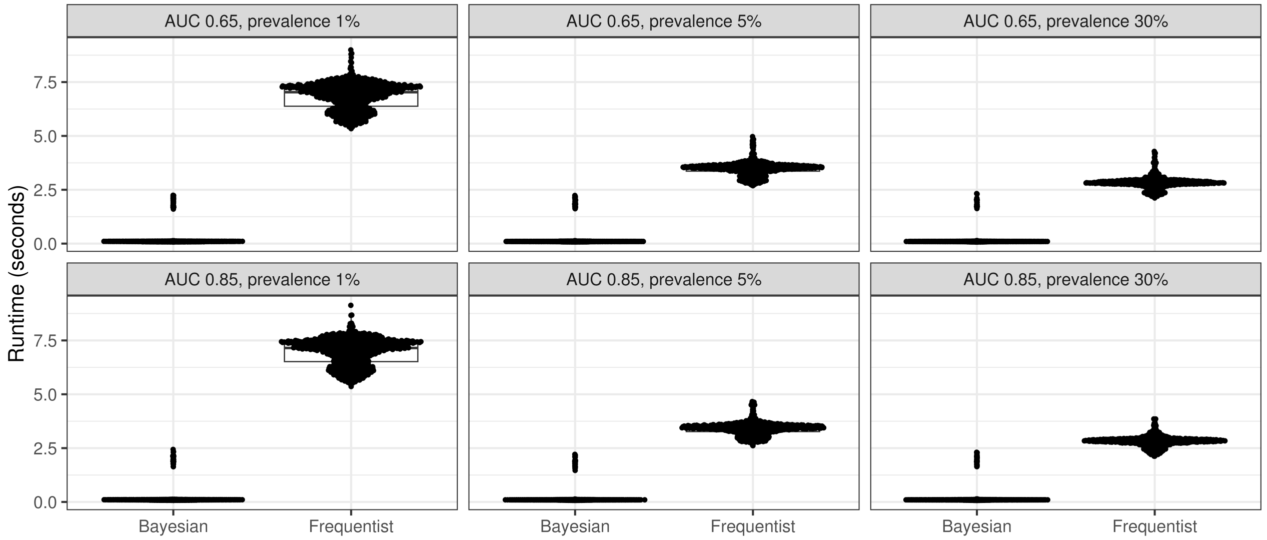

Supplementary Figure S6 **Bayesian DCA is faster than bootstrap-based Frequentist DCA for binary outcomes.** Bayesian DCA was computed using the bayesDCA R package, while the Frequentist alternative used the bootstrap-based rmda package. For each simulation run, DCA was performed for a fixed example model using a simulated test dataset of sample size corresponding to 100 expected events: 10000, 2000, and 333 observations for the 1%, 5%, and 30% prevalence settings, respectively. A total of 1000 Monte Carlo repetitions was run for each setting. The setting AUC corresponds to its maximum achievable AUC. The example model for each setting was fixed to approximate the maximum discrimination of that setting but was miscalibrated (overly extreme risk predictions). Computation time varies significantly with the overall sample size (approximately 100/prevalence) for the Frequentist case, but not in the Bayesian case.

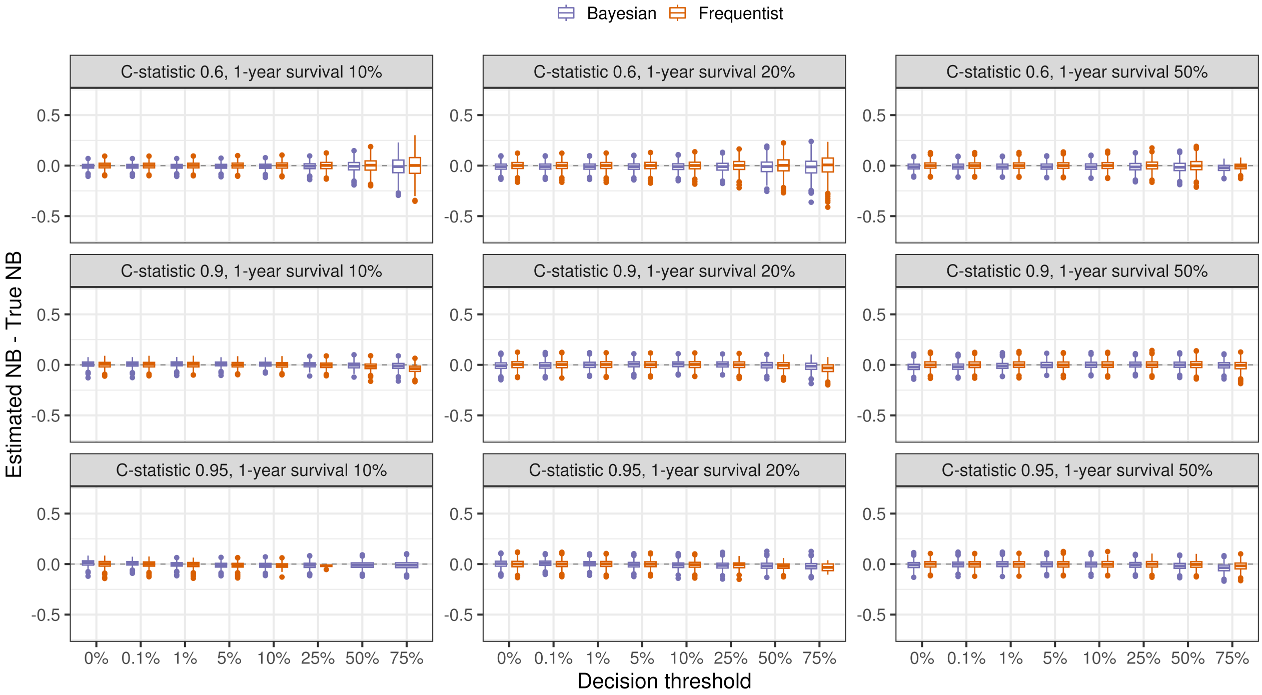

Supplementary Figure S7 **Bayesian and Frequentist DCA for survival outcomes show similar distributions of point estimate errors.** Bayesian DCA was computed using the bayesDCA R package, while the Frequentist alternative used the dcurves package. For each simulation run, DCA was performed for a fixed example model using a simulated test dataset of sample size corresponding to 100 expected events. A total of 1000 Monte Carlo repetitions was run for each setting. The setting C-statistic corresponds to its maximum achievable C-statistic. The example model for each setting was fixed to approximate the maximum discrimination of that setting but was miscalibrated (overly extreme risk predictions).

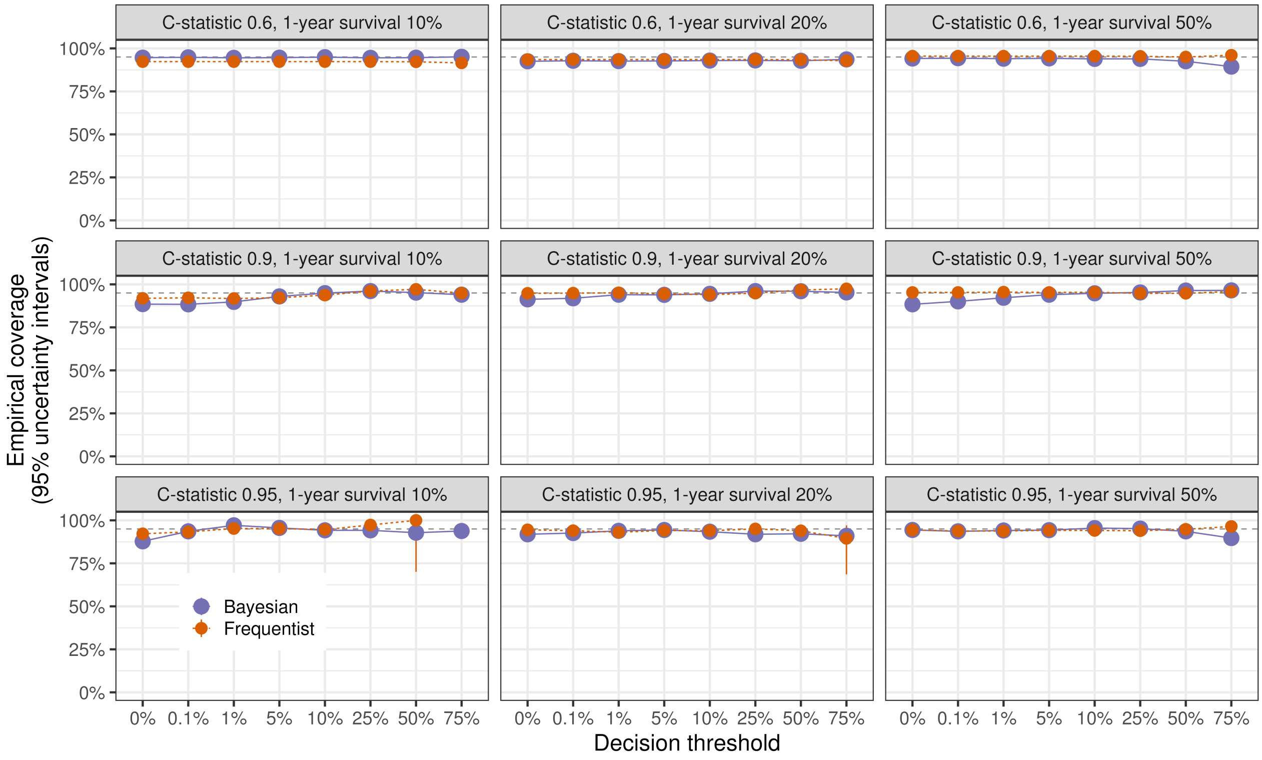

Supplementary Figure S8 **Bayesian DCA for survival outcomes shows acceptable empirical coverage**. Bayesian DCA was computed using the bayesDCA R package. For each simulation run, DCA was performed for a fixed example model using a simulated test dataset of sample size corresponding to 100 expected events. A total of 1000 Monte Carlo repetitions was run for each setting. The setting C-statistic corresponds to its maximum achievable C-statistic. The example model for each setting was fixed to approximate the maximum discrimination of that setting but was miscalibrated (overly extreme risk predictions). The Bayesian intervals show limited undercoverage and most empirical coverage values are above 90%, comparable to the observed coverage from the bootstrap-based Frequentist alternative

.

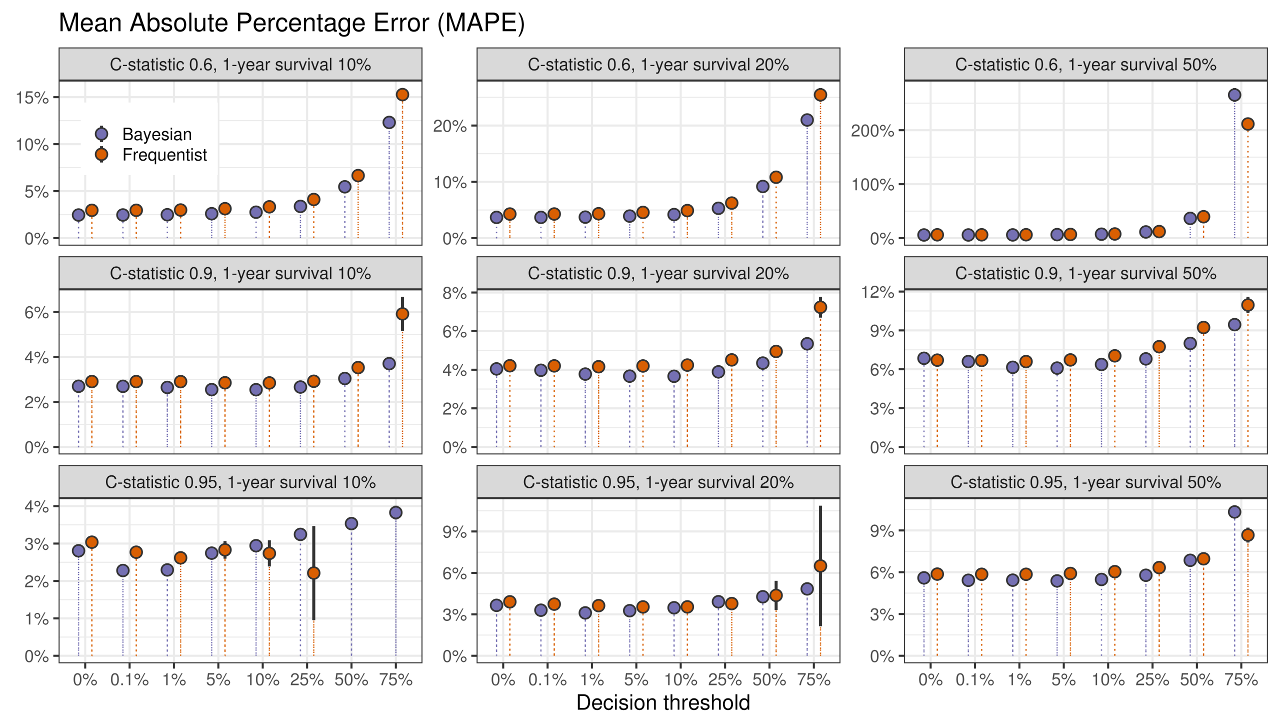

Supplementary Figure S9 **Bayesian and Frequentist DCA for survival outcomes show similar average estimation errors**. Bayesian DCA was computed using the bayesDCA R package, while the Frequentist alternative used the dcurves package. For each simulation run, DCA was performed for a fixed example model using a simulated test dataset of sample size corresponding to 100 expected events. A total of 1000 Monte Carlo repetitions was run for each setting. The setting C-statistic corresponds to its maximum achievable C-statistic. The example model for each setting was fixed to approximate the maximum discrimination of that setting but was miscalibrated (overly extreme risk predictions). Points show Mean Absolute Percentage Error (MAPE) and ranges show 95% confidence intervals. The MAPE was calculated as the average absolute difference between estimated and true NB, divided by true NB and multiplied by 100. In some simulation settings, the Frequentist method fails in the absence of observed survival times past the prediction horizon (e.g., setting with C-statistic 0.95 and 1-year survival 10%, for thresholds 50% and 75%).

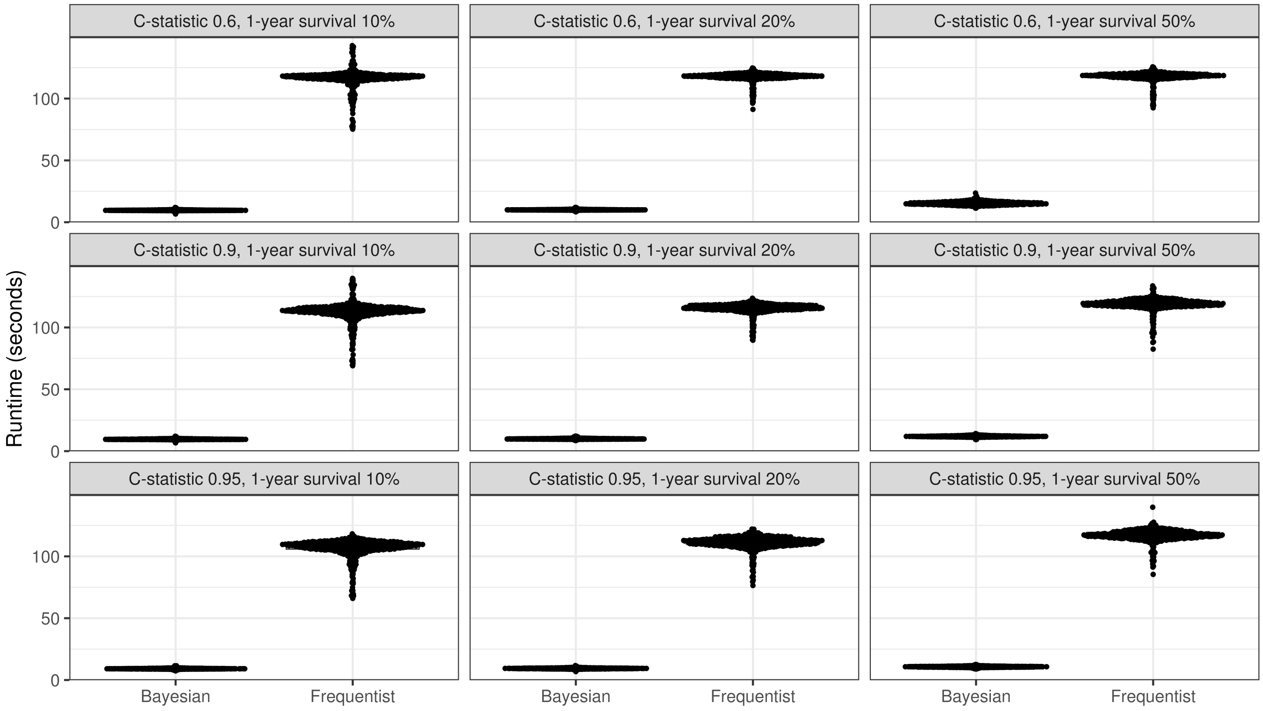

Supplementary Figure S10 **Bayesian DCA may be faster than bootstrap-based Frequentist DCA for survival outcomes.** Bayesian DCA was computed using the bayesDCA R package, while the Frequentist alternative used the dcurves package. For each simulation run, DCA was performed for a fixed example model using a simulated test dataset of sample size corresponding to 100 expected events. A total of 1000 Monte Carlo repetitions was run for each setting. The setting C-statistic corresponds to its maximum achievable C-statistic. The example model for each setting was fixed to approximate the maximum discrimination of that setting but was miscalibrated (overly extreme risk predictions). Because our custom bootstrap implementation was not optimized for speed, these results should be seen as a worst-case scenario for the Frequentist method.

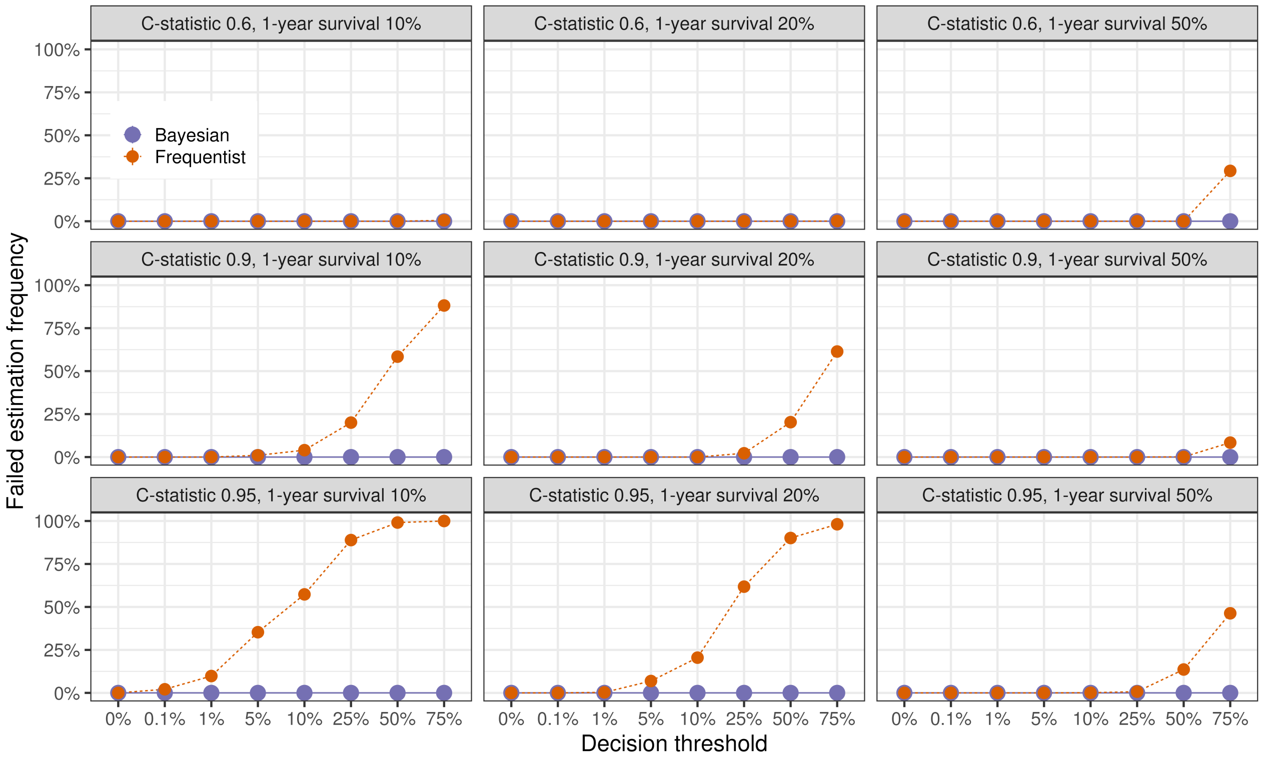

Supplementary Figure S11 **Frequentist DCA for survival outcomes fails to produce net benefit estimates in extreme settings**. Bayesian DCA was computed using the bayesDCA R package, while the Frequentist alternative used the dcurves package. For each simulation run, DCA was performed for a fixed example model using a simulated test dataset of sample size corresponding to 100 expected events. A total of 1000 Monte Carlo repetitions was run for each setting. The setting C-statistic corresponds to its maximum achievable C-statistic. The example model for each setting was fixed to approximate the maximum discrimination of that setting but was miscalibrated (overly extreme risk predictions). Points show the percentage of simulation runs in which each method produced no net benefit estimate. In some simulation settings, the Frequentist method fails in the absence of observed survival times past the prediction horizon (e.g., setting with C-statistic 0.95 and 1-year survival 10%, for thresholds 50% and 75%).

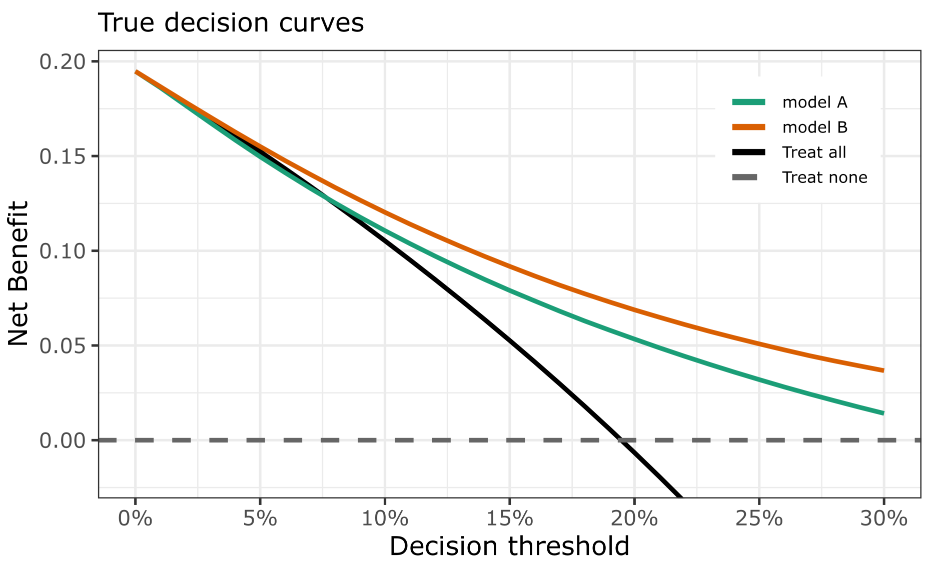

Supplementary Figure S12 **True simulated decision curves.** Decision curves were approximated with a simulated sample size of two million observations.

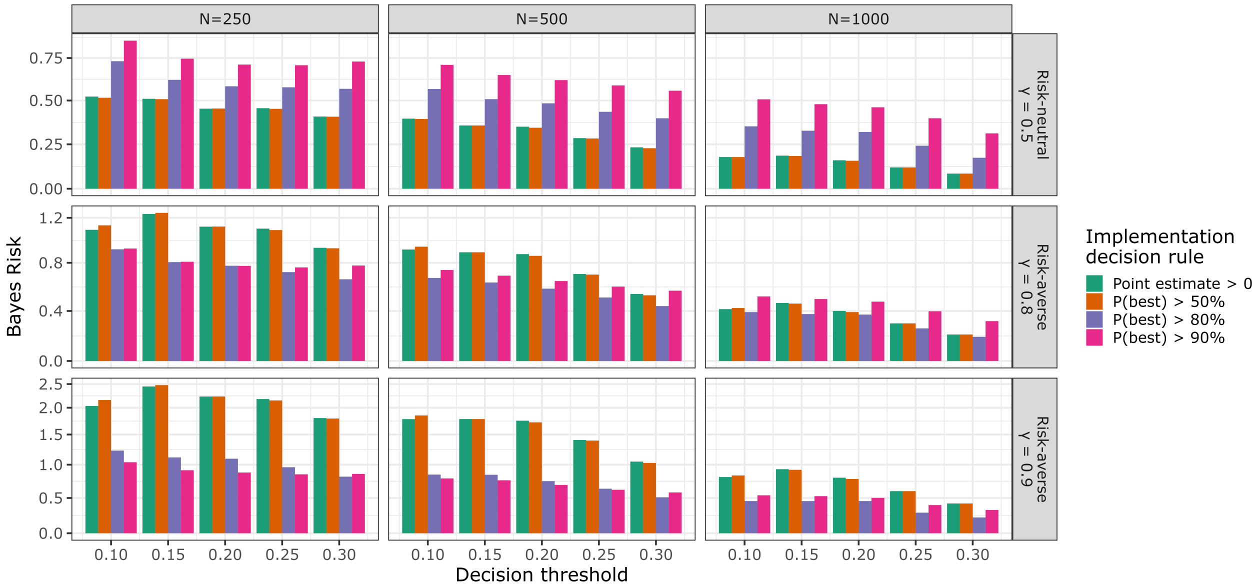

Supplementary Figure S13 **Bayes rule varies according to risk aversion.** Bayes risk according to (28) under a uniform prior for various combinations of sample size and risk aversion parameters. Sample sizes of 250, 500, and 1000 correspond to 50, 100, and 200 expected events, respectively. The baseline implementation decision rule (green) implements when the point estimate of δ_{NB} is positive. The x-axis corresponds to selected clinical decision thresholds from DCA. Bayes risk estimated from 1000 simulation runs.

Supplementary tables

Supplementary Table S1 Average performance statistics from the fixed models for each simulation setting (binary case)

Setting ID	Prevalence	Average Predicted probability	Calibration OE ratio	Calibration Slope	AUC
1	0.0102	0.0088	1.1630	0.7836	0.6525
2	0.0496	0.0565	0.8772	0.3314	0.6513
3	0.3020	0.3023	0.9990	0.3326	0.6511
4	0.0102	0.0096	1.0672	0.6668	0.8557
5	0.0546	0.0620	0.8803	0.3325	0.8460
6	0.2991	0.3242	0.9227	0.3329	0.8471

Supplementary Table S2 Average performance statistics from the fixed models for each simulation setting (survival case).

Setting ID	12-month Mortality	Average Predicted 12-month Risk	Calibration OE ratio	Calibration Slope	Concordance Index
1	0.8909	0.8903	1.0007	0.9910	0.6187
2	0.8002	0.7986	1.0019	0.9871	0.6186
3	0.5035	0.5056	0.9959	0.9949	0.6206
4	0.8971	0.8510	1.0543	0.8022	0.8889
5	0.7911	0.7478	1.0579	0.7994	0.8921
6	0.4905	0.4930	0.9950	0.8005	0.9053
7	0.8970	0.8459	1.0604	0.8011	0.9496
8	0.8087	0.7584	1.0664	0.7992	0.9517
9	0.5254	0.5200	1.0105	0.7992	0.9605

Supplementary Table S3 Summary of informative Beta priors used in EVPI simulation.

	Sensitivity				Specificity			
Threshold	Mean	95% Cr.I.	Shape 1	Shape 2	Mean	95% Cr.I.	Shape 1	Shape 2
0.01	0.95	(0.76 — 1.00)	9.5	0.5	0.05	(0.00 — 0.24)	0.5	9.5
0.02	0.90	(0.66 — 1.00)	9.0	1.0	0.10	(0.00 — 0.34)	1.0	9.0
0.05	0.75	(0.46 — 0.95)	7.5	2.5	0.25	(0.05 — 0.54)	2.5	7.5
0.1	0.50	(0.21 — 0.79)	5.0	5.0	0.50	(0.21 — 0.79)	5.0	5.0

*Prior sample size was fixed at 10.

**A uniform Beta(1, 1) prior was used for prevalence.
