# Supplementary material for: Bayesian Decision Curve Analysis With Bayesdca
Source: Stat Med. 2024 Dec 1;43(30):6042–58. doi: 10.1002/sim.10277 (PMC11639651; doi:10.1002/sim.10277)
Supplement: Supplementary file 1 — Appendix S1. Supplementary methods. [file SIM-43-6042-s001.zip › sim10277-sup-0003-DataS1.zip!/bayesdca-5295e3c320f747cf50839b2aa3ddae936e69f45e/docs/articles/survival-outcomes.html]

Survival outcomes • bayesDCA


     

 


Skip to contents


bayesDCA
0.0.0.9000


- Reference
- Articles

  Survival outcomes

# Survival outcomes

Source: `vignettes/survival-outcomes.Rmd`

`survival-outcomes.Rmd`

To illustrate Bayesian DCA on survival data, we load the example
dataset `dca_survival_data` shown below.

```
library(bayesDCA)
head(dca_survival_data)
#>   outcomes.time outcomes.status model_predictions binary_test
#> 1     0.2948349       0.0000000        0.18806301           0
#> 2     1.8747960       0.0000000        0.25349061           1
#> 3     0.0355183       0.0000000        0.01025269           0
#> 4     1.2850960       0.0000000        0.04836840           0
#> 5     0.2398763       0.0000000        0.23502042           1
#> 6     2.0934150       0.0000000        0.04611183           0
```

The dataset contains the time-to-event `outcomes` (a
`survival::Surv` object), the predictions from a prognostic
model, and the results from a binary prognostic test. The time horizon
for the event prediction is one time units (e.g., year), so we set
`prediction_time = 1`. We also set `chains = 1` to
speed up MCMC sampling with Stan (in
practice you should use at least `chains = 4`, maybe with
`cores = 4` for speed as well).

```
fit <- dca_surv(dca_survival_data, prediction_time = 1, iter = 1000, chains = 1)
plot(fit)
```

## Interrogating the output

We can do all kinds of output interrogation just like with binary
outcomes.

### What is the best decision strategy?

```
compare_dca(fit)
```

### Are the prediction model or the binary test useful at all?

```
compare_dca(fit, type = "useful")
```

### Is the model better than the test?

```
compare_dca(fit, 
            strategies = c("model_predictions", "binary_test"), 
            type = "pairwise")
```

### What is the price of uncertainty?

```
compare_dca(fit, .evpi = TRUE)
```

## On this page

Developed by Giuliano Netto Flores Cruz.

Site built with pkgdown 2.0.7.

Using preferably template.
