# Supplementary material for: Bayesian Decision Curve Analysis With Bayesdca
Source: Stat Med. 2024 Dec 1;43(30):6042–58. doi: 10.1002/sim.10277 (PMC11639651; doi:10.1002/sim.10277)
Supplement: Supplementary file 1 — Appendix S1. Supplementary methods. [file SIM-43-6042-s001.zip › sim10277-sup-0003-DataS1.zip!/bayesdca-5295e3c320f747cf50839b2aa3ddae936e69f45e/docs/authors.html]

 Authors and Citation • bayesDCA         
Skip to contents


bayesDCA
0.0.0.9000


- Reference
- Articles

  Survival outcomes

# Authors and Citation

## Authors

- **Giuliano Netto Flores Cruz**. Author, maintainer.

## Citation

Source: `inst/CITATION`

Cruz, GNF (2022). Bayesian Decision Curve Analysis with bayesDCA. R package version 0.0.0.9000. URL https://giulianonetto.github.io/bayesdca.

```
@Manual{,
  title = {Bayesian Decision Curve Analysis with {bayesDCA}},
  author = {Giuliano Netto Flores Cruz},
  year = {2022},
  url = {https://giulianonetto.github.io/bayesdca/},
  note = {R package version 0.0.0.9000},
}
```

## On this page

Developed by Giuliano Netto Flores Cruz.

Site built with pkgdown 2.0.7.

Using preferably template.
