# Supplementary material for: Bayesian Decision Curve Analysis With Bayesdca
Source: Stat Med. 2024 Dec 1;43(30):6042–58. doi: 10.1002/sim.10277 (PMC11639651; doi:10.1002/sim.10277)
Supplement: Supplementary file 1 — Appendix S1. Supplementary methods. [file SIM-43-6042-s001.zip › sim10277-sup-0003-DataS1.zip!/bayesdca-5295e3c320f747cf50839b2aa3ddae936e69f45e/docs/index.html]

Bayesian Decision Curve Analysis • bayesDCA


     

 


Skip to contents


bayesDCA
0.0.0.9000


- Reference
- Articles

  Survival outcomes

# bayesDCA

Perform Bayesian Decision Curve Analysis for clinical prediction models and diagnostic tests.

When validating a clinical prediction model, you may end up with AUC 0.79 and a slight miscalibration. How do you know if that is good enough for your model to be clinically useful? The same question can be asked if you have, for instance, a binary diagnostic test with sensitivity of 75% and specificity of 68%. Decision Curve Analysis helps us find an answer - see Vickers, van Calster & Steyerberg, 2019 for an introduction to DCA. Here, we use Bayesian methods to accurately quantify uncertainty in our decisions curves - powered by Stan.

# Installation

You can install the development version of bayesDCA from GitHub with:

```
# install.packages("devtools")
devtools::install_github("giulianonetto/bayesdca")
```

# Running Bayesian DCA

You can use `bayesDCA` to evaluate predictive models as well as binary tests.

All you need is a `data.frame` with a column named `outcomes` (0 or 1) and one column for each model or test being evaluated. In the example below, the `PredModelData` includes the probability predictions from a model (`"predictions" column`) and the results from a binary test (`"binary_test"` column). The names of these columns don’t matter (except for the `outcomes` column, which should always be present).

```
library(bayesDCA)
data(PredModelData)
head(PredModelData)
#>   outcomes predictions binary_test
#> 1        0  0.01280653           0
#> 2        0  0.13981948           0
#> 3        0  0.03566458           0
#> 4        0  0.02351731           0
#> 5        0  0.00863298           0
#> 6        0  0.00959754           0
```

Run the DCA itself:

```
fit <- dca(PredModelData)
plot(fit)
```

# Interpreting Bayesian DCA

Each decision curve represents the predictive performance of a decision strategy – yes, treating all (or none) is a valid decision strategy. Given the estimated decision curves, we may want to ask several questions, such as:

- What is the best decision strategy?
- Are the strategies based on predictive models or tests useful *at all*?
- Is the predictive model guaranteed to be better than the binary test?
- What is the consequence of the uncertainty imposed by the available data?

The `bayesDCA` R package provides an easy workflow for answering these questions. In particular, the “DCA part” of Bayesian DCA allows answering the first three questions; adding the “Bayesian part” allows quantifying uncertainty around those answers.

The best decision strategy is the one that maximizes the observed net benefit. However, if the uncertainty is large, more data may be necessary to increase confidence in our conclusions. The fourth question directly quantifies what we might lose, in terms of net benefit, by drawing conclusions under the current level of uncertainty.

### What is the best decision strategy?

Say you want to infer what is the best decision strategy among the prediction model, the binary test, and the default strategies (treat all/none). You can then compare the decision curves:

```
compare_dca(fit, type = "best")
```

For most decision thresholds, the model predictions maximize the observed net benefit (i.e., have the highest posterior means) and hence is the best decision strategy given the available data. However, notice that uncertainty is quite large at least for thresholds below 30%, where conclusions might be noisy and more data could be beneficial.

### Are the prediction model or the binary test useful at all?

Well, maybe not everyone has access to both the prediction model and the binary test. But is each of them useful at all? Here, “useful” means better than the default strategies (treat all/none), following the definition in Wynants et al. (2018).

```
compare_dca(fit, type = "useful")
```

### Is the model better than the test?

We can also do pairwise comparisons by specifying the pair of strategies we want to compare with the `models_or_tests` argument (set it to a single value to compare it against default strategies).

```
compare_dca(fit, 
            strategies = c("predictions", "binary_test"), 
            type = "pairwise")
```

### What is the consequence of uncertainty?

To answer that, we need an estimate of the *Expected Value of Perfect Information* (EVPI) for model validation as defined in Sadatsafavi et al. (2022). You may think of this as the price or consequence of uncertainty. Since we only have a sample dataset, we don’t know for sure what is the best decision strategy for each threshold. The EVPI quantifies the expected loss in net benefit associated with that uncertainty. If it is high for a given threshold, then we would greatly benefit from more data to validate our decision strategies.

```
compare_dca(fit, .evpi = TRUE)
```

Notice that the EVPI follows closely the uncertainty around the best strategy: the larger the uncertainty, the more we are expected to benefit from accessing “perfect information”.

# Using external information to estimate prevalence

Say you are validating tests using a nested case-control study, so the prevalence parameter must come from the larger sample from which cases and controls were selected. Another example is when you want to use an external estimate of prevalence (say from a large prospective cross-sectional study). You can do so by passing the `external_prevalence_data` argument to `dca`.

```
fit <- dca(PredModelData,
           external_prevalence_data = c(100, 500))
plot(fit)
```

Notice also that the `external_prevalence_data` information is used only to estimate the prevalence, which is then used in the net benefit calculation. Sensitivity and specificity use the original information in `PredModelData`.

## Links

- Browse source code

## License

- Full license
- GPL (>= 3)

## Citation

- Citing bayesDCA

## Developers

- Giuliano Netto Flores Cruz   
   Author, maintainer

## Dev status

Developed by Giuliano Netto Flores Cruz.

Site built with pkgdown 2.0.7.

Using preferably template.
