# Supplementary material for: Bayesian Decision Curve Analysis With Bayesdca
Source: Stat Med. 2024 Dec 1;43(30):6042–58. doi: 10.1002/sim.10277 (PMC11639651; doi:10.1002/sim.10277)
Supplement: Supplementary file 1 — Appendix S1. Supplementary methods. [file SIM-43-6042-s001.zip › sim10277-sup-0003-DataS1.zip!/bayesdca-5295e3c320f747cf50839b2aa3ddae936e69f45e/docs/reference/Docking2021Data.html]

 Survival data from Docking, 2021 - Figures 3B-3E — Docking2021Data • bayesDCA         
Skip to contents


bayesDCA
0.0.0.9000


- Reference
- Articles

  Survival outcomes

# Survival data from Docking, 2021 - Figures 3B-3E

Source: `R/data.R`

`Docking2021Data.Rd`

A dataset containing the survival data from Docking, 2021.
Dataset includes selected data used for Figures 3B to 3E.
Observations with missing survival time (in weeks)
or missing survival events were excluded.
AML prognostic score (APS) values were kept as an example of continuous biomarker.

## Usage

```
Docking2021Data
```

## Format

A data frame with 635 rows and 5 variables:

fig\_id
:   Character indicating corresponding figure in the original paper.

aps\_lasso\_score
:   APS value

aps\_lasso\_class
:   APS tercile computed within study cohort.

survival\_event
:   Death is 1, censored/slive is 0.

survival\_weeks
:   Overall survival in weeks.
...

## Source

https://giulianonetto.github.io/bayesdca/

## On this page

Developed by Giuliano Netto Flores Cruz.

Site built with pkgdown 2.0.7.

Using preferably template.
