# Supplementary material for: Bayesian Decision Curve Analysis With Bayesdca
Source: Stat Med. 2024 Dec 1;43(30):6042–58. doi: 10.1002/sim.10277 (PMC11639651; doi:10.1002/sim.10277)
Supplement: Supplementary file 1 — Appendix S1. Supplementary methods. [file SIM-43-6042-s001.zip › sim10277-sup-0003-DataS1.zip!/bayesdca-5295e3c320f747cf50839b2aa3ddae936e69f45e/docs/reference/PredModelData.html]

 Predictive Model Evaluation Data. — PredModelData • bayesDCA         
Skip to contents


bayesDCA
0.0.0.9000


- Reference
- Articles

  Survival outcomes

# Predictive Model Evaluation Data.

Source: `R/data.R`

`PredModelData.Rd`

A dataset containing the outcomes (0 or 1), probability predictions
for an example model, and results from an example binary test.
Binary test generated using sensitivity
and specificity of 80%. Based on `rmda::dcaData`.

## Usage

```
PredModelData
```

## Format

A data frame with 500 rows and 3 variables:

outcomes
:   binary outcomes (0 or 1, int.)

predictions
:   predicted probabilities (num.)

binary\_test
:   binary test results (0 or 1, int.)
...

## Source

https://giulianonetto.github.io/bayesdca/

## On this page

Developed by Giuliano Netto Flores Cruz.

Site built with pkgdown 2.0.7.

Using preferably template.
