# Supplementary material for: Bayesian Decision Curve Analysis With Bayesdca
Source: Stat Med. 2024 Dec 1;43(30):6042–58. doi: 10.1002/sim.10277 (PMC11639651; doi:10.1002/sim.10277)
Supplement: Supplementary file 1 — Appendix S1. Supplementary methods. [file SIM-43-6042-s001.zip › sim10277-sup-0003-DataS1.zip!/bayesdca-5295e3c320f747cf50839b2aa3ddae936e69f45e/docs/reference/bayesDCA-package.html]

 The 'bayesDCA' package. — bayesDCA-package • bayesDCA         
Skip to contents


bayesDCA
0.0.0.9000


- Reference
- Articles

  Survival outcomes

# The 'bayesDCA' package.

Source: `R/bayesDCA-package.R`

`bayesDCA-package.Rd`

A DESCRIPTION OF THE PACKAGE

## References

Stan Development Team (2020). RStan: the R interface to Stan. R package version 2.21.2. https://mc-stan.org

Developed by Giuliano Netto Flores Cruz.

Site built with pkgdown 2.0.7.

Using preferably template.
