# Supplementary material for: Bayesian Decision Curve Analysis With Bayesdca
Source: Stat Med. 2024 Dec 1;43(30):6042–58. doi: 10.1002/sim.10277 (PMC11639651; doi:10.1002/sim.10277)
Supplement: Supplementary file 1 — Appendix S1. Supplementary methods. [file SIM-43-6042-s001.zip › sim10277-sup-0003-DataS1.zip!/bayesdca-5295e3c320f747cf50839b2aa3ddae936e69f45e/docs/reference/dca.html]

 Bayesian Decision Curve Analysis for Predictive Models and Binary tests — dca • bayesDCA         
Skip to contents


bayesDCA
0.0.0.9000


- Reference
- Articles

  Survival outcomes

# Bayesian Decision Curve Analysis for Predictive Models and Binary tests

Source: `R/dca.R`

`dca.Rd`

Estimate decision curves for a list of predictive models and/or
binary tests all at once. Necessary to make comparative inferences across
multiple models or tests
using their corresponding posterior draws.

## Usage

```
dca(
  .data,
  thresholds = seq(0, 0.5, length = 51),
  prior_p = NULL,
  prior_se = NULL,
  prior_sp = NULL,
  priors = NULL,
  threshold_varying_prior = FALSE,
  ignorance_region_cutpoints = c(0.25, 0.75) * max(thresholds),
  min_sens_prior_mean = 0.01,
  max_sens_prior_mean = 0.99,
  max_sens_prior_sample_size = 5,
  ignorance_region_mean = 0.5,
  ignorance_region_sample_size = 2,
  prev_prior_mean = 0.5,
  prev_prior_sample_size = 2,
  summary_probs = c(0.025, 0.975),
  external_prevalence_data = NULL,
  prior_only = FALSE,
  n_draws = 4000
)
```

## Arguments

.data
:   A data.frame with an `outcomes` column (0 or 1 for each individual)
    and one or more columns with predicted probabilities from each of desired list
    of predictive models, or with 0 or 1 indicator from each of desired list of
    binary tests.

thresholds
:   Numeric vector with probability thresholds with which
    the net benefit should be computed (default is `seq(0, 0.5, length = 51)`).

prior\_p, prior\_se, prior\_sp
:   Non-negative shape values for
    Beta(alpha, beta) priors used for p, Se, and Sp, respectively.
    Default is uniform prior for all parameters - Beta(1, 1).
    A single vector of the form `c(a, b)` can be provided for each.

priors
:   A list with threshold- and model-specific priors
    should contain a vector for shape1 of prevalence (named `p1`)
    and shape2 (named `p2`). Similarly for Se1/Se2 and Sp1/Sp2, except
    these should be matrices with as many rows as thresholds and
    as many columns as models or tests.

summary\_probs
:   Probabilities used to compute credible
    intervals (defaults to a 95% Cr.I.).

external\_prevalence\_data
:   Vector with two positive
    integers giving number of diseased and
    non-diseased individuals, respectively, from external data
    (e.g., if analyzing nested case-control data,
    this is the number of cases and non-cases in the source
    population).

prior\_only
:   If set to TRUE, will produce prior DCA.

keep\_fit
:   Logical indicating whether to keep `stanfit` in
    the output (default is FALSE).

keep\_draws
:   Logical indicating whether to keep posterior
    draws from `stanfit` object (default is TRUE).

constant\_prior
:   If TRUE (default), it will set a single
    prior for all models or tests in all thresholds.
    If FALSE, the prior will be threshold and, potentially, model/test-specific.

min\_prior\_mean, max\_prior\_mean
:   Minimum
    and maximum prior mean for sensitivity and specificity.
    Only used if `constant_prior = FALSE`.

refresh
:   Control verbosity of `rstan::sampling`
    (check its help
    page for details).

## Value

An object of class `BayesDCA`

## Examples

```
data(PredModelData)
fit <- dca(PredModelData)
plot(fit)
```

## On this page

Developed by Giuliano Netto Flores Cruz.

Site built with pkgdown 2.0.7.

Using preferably template.
