# Supplementary material for: Bayesian Decision Curve Analysis With Bayesdca
Source: Stat Med. 2024 Dec 1;43(30):6042–58. doi: 10.1002/sim.10277 (PMC11639651; doi:10.1002/sim.10277)
Supplement: Supplementary file 1 — Appendix S1. Supplementary methods. [file SIM-43-6042-s001.zip › sim10277-sup-0003-DataS1.zip!/bayesdca-5295e3c320f747cf50839b2aa3ddae936e69f45e/docs/reference/dca_surv.html]

 Bayesian Decision Curve Analysis
for Survival outcomes — dca\_surv • bayesDCA         
Skip to contents


bayesDCA
0.0.0.9000


- Reference
- Articles

  Survival outcomes

# Bayesian Decision Curve Analysis for Survival outcomes

Source: `R/dca_surv.R`

`dca_surv.Rd`

Bayesian Decision Curve Analysis
for Survival outcomes

## Usage

```
dca_surv(
  .data,
  prediction_time,
  thresholds = seq(0, 0.5, length = 51),
  keep_draws = TRUE,
  keep_fit = FALSE,
  summary_probs = c(0.025, 0.975),
  positivity_prior = c(1, 1),
  shape_prior = c("student", "gamma"),
  scale_prior = c("student", "gamma"),
  shape_prior_pars = c(10, 0, 1.5),
  scale_prior_pars = c(30, 0, 100),
  prior_only = FALSE,
  iter = 4000,
  refresh = 0,
  ...
)
```

## Arguments

.data
:   dataframe whose first column named "outcomes" is a `survival::Surv` object
    and remaining columns are the decision strategies to assess.

prediction\_time
:   Prediction time horizon (e.g., if models predict risk
    of death at one year and data is in year, `prediction_time` should be `1`.)

thresholds
:   Decision thresholds -- within interval (0, 1).

keep\_draws
:   If true, posterior draws are kept in the output object.

keep\_fit
:   If true, `stanfit` object is kept in the output object.

summary\_probs
:   Probabilities for posterior credible intervals (defaults to a 95% Cr.I.).

positivity\_prior
:   Shape parameters for prior on positivity probability.

shape\_prior
:   type of prior distribution for shape parameter of the Weibull distribution. Either "student" or "gamma".

scale\_prior
:   type of prior distribution for scale parameter of the Weibull distribution. Either "student" or "gamma".

shape\_prior\_pars
:   vector with prior parameters for the prior shape of the Weibull distribution.
    If `shape_prior="student"`, it should be a vector of length 3 with degrees of freedom, mean, and scale,
    respectively; if `shape_prior="gamma"`, it should be a vector of length 2 with shape and rate, respectively.

prior\_only
:   If TRUE, samples from the prior only.

iter
:   Passed to `rstan::sampling`. Number of iterations/draws for Stan.

refresh
:   Controls verbosity of
    `rstan::sampling`

...
:   Arguments passed to
    `rstan::sampling` (e.g. iter, chains). # nolint

## Value

An object of class `BayesDCASurv`

## Examples

```
if (FALSE) {
data(dca_survival_data)
fit <- dca_surv(dca_survival_data, prediction_time = 1, iter = 1000, chains = 1)
plot(fit)
}
```

## On this page

Developed by Giuliano Netto Flores Cruz.

Site built with pkgdown 2.0.7.

Using preferably template.
