# Supplementary material for: Bayesian Decision Curve Analysis With Bayesdca
Source: Stat Med. 2024 Dec 1;43(30):6042–58. doi: 10.1002/sim.10277 (PMC11639651; doi:10.1002/sim.10277)
Supplement: Supplementary file 1 — Appendix S1. Supplementary methods. [file SIM-43-6042-s001.zip › sim10277-sup-0003-DataS1.zip!/bayesdca-5295e3c320f747cf50839b2aa3ddae936e69f45e/docs/reference/dot-dca_binary.html]

 Fit Bayesian Decision Curve Analysis using
Stan for list of models or binary tests — .dca\_binary • bayesDCA         
Skip to contents


bayesDCA
0.0.0.9000


- Reference
- Articles

  Survival outcomes

# Fit Bayesian Decision Curve Analysis using Stan for list of models or binary tests

Source: `R/dca.R`

`dot-dca_binary.Rd`

Fit Bayesian Decision Curve Analysis using
Stan for list of models or binary tests

## Usage

```
.dca_binary(
  n_thr,
  strategies,
  N,
  d,
  tp,
  tn,
  thresholds,
  prior_p1,
  prior_p2,
  prior_Se1,
  prior_Se2,
  prior_Sp1,
  prior_Sp2,
  N_ext = 0,
  d_ext = 0,
  n_draws = 4000
)
```

## Arguments

n\_thr
:   Number of thresholds (int.).

N
:   Sample size (vector of integers of length `n_thr`).

d
:   Diseased: number of diseased persons or
    events (vector of integers of length `n_thr`).

tp
:   True Positives: number of diseased persons correctly
    identified as such by the diagnostic test of prediction
    model (matrix of integers of size `n_thr` by `n_strategies`).

tn
:   True Negatives: number of diseased persons correctly
    identified as such by the diagnostic test of prediction
    model (matrix of integers of size `n_thr` by
    `n_strategies`).

thresholds
:   Numeric vector with probability thresholds with which
    the net benefit should be computed (default is `seq(0.01, 0.5, 0.01)`).

N\_ext, d\_ext
:   External sample size and number of
    diseased individuals (or cases), respectively, used to
    adjust prevalence.

n\_strategies
:   Number of models or binary tests (int.).

prior\_p, prior\_se, prior\_sp
:   Prior parameters for
    prevalence, sensitivity, and specificity (numeric matrices
    of size `n_thr` by `n_strategies`).

refresh
:   Control verbosity of
    `rstan::sampling`.

...
:   Arguments passed to
    `rstan::sampling` (e.g. iter, chains).

## Value

An object of class
`stanfit` returned by `rstan::sampling` (e.g. iter, chains)

## On this page

Developed by Giuliano Netto Flores Cruz.

Site built with pkgdown 2.0.7.

Using preferably template.
