# Supplementary material for: Bayesian Decision Curve Analysis With Bayesdca
Source: Stat Med. 2024 Dec 1;43(30):6042–58. doi: 10.1002/sim.10277 (PMC11639651; doi:10.1002/sim.10277)
Supplement: Supplementary file 1 — Appendix S1. Supplementary methods. [file SIM-43-6042-s001.zip › sim10277-sup-0003-DataS1.zip!/bayesdca-5295e3c320f747cf50839b2aa3ddae936e69f45e/docs/reference/dot-dca_stan_surv.html]

 Fit Bayesian Decision Curve Analysis using Stan for survival outcomes — .dca\_stan\_surv • bayesDCA         
Skip to contents


bayesDCA
0.0.0.9000


- Reference
- Articles

  Survival outcomes

# Fit Bayesian Decision Curve Analysis using Stan for survival outcomes

Source: `R/dca_surv.R`

`dot-dca_stan_surv.Rd`

Fit Bayesian Decision Curve Analysis using Stan for survival outcomes

## Usage

```
.dca_stan_surv(
  n_thr,
  n_models_or_tests,
  n_intervals,
  thresholds,
  time_exposed,
  posterior_alpha,
  posterior_beta,
  posterior_alpha0,
  posterior_beta0,
  pos_post1,
  pos_post2,
  other_models_indices,
  refresh = 0,
  ...
)
```

## Arguments

refresh
:   Control verbosity of `rstan::sampling`.

...
:   Arguments passed to `rstan::sampling` (e.g. iter, chains).

## Value

An object of class `stanfit` returned by `rstan::sampling` (e.g. iter, chains)

## On this page

Developed by Giuliano Netto Flores Cruz.

Site built with pkgdown 2.0.6.

Using preferably template.
