# Supplementary material for: Bayesian Decision Curve Analysis With Bayesdca
Source: Stat Med. 2024 Dec 1;43(30):6042–58. doi: 10.1002/sim.10277 (PMC11639651; doi:10.1002/sim.10277)
Supplement: Supplementary file 1 — Appendix S1. Supplementary methods. [file SIM-43-6042-s001.zip › sim10277-sup-0003-DataS1.zip!/bayesdca-5295e3c320f747cf50839b2aa3ddae936e69f45e/docs/reference/dot-dca_stan_surv_weibull.html]

 Fit Bayesian Decision Curve Analysis
using Stan for survival outcomes (Weibull model) — .dca\_stan\_surv\_weibull • bayesDCA         
Skip to contents


bayesDCA
0.0.0.9000


- Reference
- Articles

  Survival outcomes

# Fit Bayesian Decision Curve Analysis using Stan for survival outcomes (Weibull model)

Source: `R/dca_surv.R`

`dot-dca_stan_surv_weibull.Rd`

Fit Bayesian Decision Curve Analysis
using Stan for survival outcomes (Weibull model)

## Usage

```
.dca_stan_surv_weibull(
  sample_size,
  n_thr,
  n_strategies,
  event_times_stacked,
  censored_times_stacked,
  n_event_times_stacked,
  n_censored_times_stacked,
  event_times_start_positions,
  censored_times_start_positions,
  event_times_sizes,
  censored_times_sizes,
  prediction_time,
  thresholds,
  pos_post1,
  pos_post2,
  total_event_times,
  total_censored_times,
  event_times_marginal,
  censored_times_marginal,
  other_models_indices,
  prior_only,
  shape_prior,
  scale_prior,
  shape_prior_pars,
  scale_prior_pars,
  iter = 2000,
  refresh = 0,
  ...
)
```

## Arguments

refresh
:   Control verbosity of
    `rstan::sampling`. # nolint

...
:   Arguments passed to
    `rstan::sampling` (e.g. iter, chains). # nolint

## Value

An object of class
`stanfit` returned by `rstan::sampling` (e.g. iter, chains) # nolint

## On this page

Developed by Giuliano Netto Flores Cruz.

Site built with pkgdown 2.0.7.

Using preferably template.
