# Supplementary material for: Bayesian Decision Curve Analysis With Bayesdca
Source: Stat Med. 2024 Dec 1;43(30):6042–58. doi: 10.1002/sim.10277 (PMC11639651; doi:10.1002/sim.10277)
Supplement: Supplementary file 1 — Appendix S1. Supplementary methods. [file SIM-43-6042-s001.zip › sim10277-sup-0003-DataS1.zip!/bayesdca-5295e3c320f747cf50839b2aa3ddae936e69f45e/docs/reference/dot-extract_dca_surv_draws.html]

 Get posterior draws from time-to-event DCA stanfit — .extract\_dca\_surv\_draws • bayesDCA         
Skip to contents


bayesDCA
0.0.0.9000


- Reference
- Articles

  Survival outcomes

# Get posterior draws from time-to-event DCA stanfit

Source: `R/dca_surv.R`

`dot-extract_dca_surv_draws.Rd`

Get posterior draws from time-to-event DCA stanfit

## Usage

```
.extract_dca_surv_draws(fit, strategies)
```

## Arguments

fit
:   A stanfit object.

strategies
:   Vector of names of models
    or binary tests under assessment.

## On this page

Developed by Giuliano Netto Flores Cruz.

Site built with pkgdown 2.0.7.

Using preferably template.
