# Supplementary material for: Bayesian Decision Curve Analysis With Bayesdca
Source: Stat Med. 2024 Dec 1;43(30):6042–58. doi: 10.1002/sim.10277 (PMC11639651; doi:10.1002/sim.10277)
Supplement: Supplementary file 1 — Appendix S1. Supplementary methods. [file SIM-43-6042-s001.zip › sim10277-sup-0003-DataS1.zip!/bayesdca-5295e3c320f747cf50839b2aa3ddae936e69f45e/docs/reference/dot-extract_dca_surv_summary.html]

 Extract posterior summaries for BayesDCASurv object — .extract\_dca\_surv\_summary • bayesDCA         
Skip to contents


bayesDCA
0.0.0.9000


- Reference
- Articles

  Survival outcomes

# Extract posterior summaries for `BayesDCASurv` object

Source: `R/dca_surv.R`

`dot-extract_dca_surv_summary.Rd`

Extract posterior summaries for `BayesDCASurv` object

## Usage

```
.extract_dca_surv_summary(fit, summary_probs, thresholds, strategies)
```

## Arguments

summary\_probs
:   Probabilities for posterior credible intervals (defaults to a 95% Cr.I.).

thresholds
:   Decision thresholds -- within interval (0, 1).

strategies
:   Vector of names of models or binary tests under assessment.

## Value

An object of class `BayesDCASurv`

## On this page

Developed by Giuliano Netto Flores Cruz.

Site built with pkgdown 2.0.7.

Using preferably template.
