# Supplementary material for: Bayesian Decision Curve Analysis With Bayesdca
Source: Stat Med. 2024 Dec 1;43(30):6042–58. doi: 10.1002/sim.10277 (PMC11639651; doi:10.1002/sim.10277)
Supplement: Supplementary file 1 — Appendix S1. Supplementary methods. [file SIM-43-6042-s001.zip › sim10277-sup-0003-DataS1.zip!/bayesdca-5295e3c320f747cf50839b2aa3ddae936e69f45e/docs/reference/dot-get_nonconstant_prior_parameters.html]

 Get threshold- and model-specific priors for Bayesian DCA — .get\_nonconstant\_prior\_parameters • bayesDCA         
Skip to contents


bayesDCA
0.0.0.9000


- Reference
- Articles

  Survival outcomes

# Get threshold- and model-specific priors for Bayesian DCA

Source: `R/utils.R`

`dot-get_nonconstant_prior_parameters.Rd`

Get threshold- and model-specific priors for Bayesian DCA

## Usage

```
.get_nonconstant_prior_parameters(
  thresholds,
  n_models_or_tests,
  shift = 0.45,
  slope = 0.025,
  prior_sample_size = NULL,
  min_prior_sample_size = 20,
  max_prior_sample_size = 100,
  slope_prior_sample_size = 300,
  prior_p = NULL,
  min_mean_se = 0.1,
  max_mean_se = 0.9,
  min_mean_sp = 0.1,
  max_mean_sp = 0.9
)
```

## Arguments

n\_models\_or\_tests
:   Number of models or tests (int.).

prior\_sample\_size
:   Prior sample size or strength.

min\_prior\_sample\_size
:   Minimum prior sample size or strength.

max\_prior\_sample\_size
:   Maximum prior sample size or strength.

slope\_prior\_sample\_size
:   Rate of change in prior sample size or strength.

prior\_p, prior\_se, prior\_sp
:   Non-negative shape values for
    Beta(alpha, beta) priors used for p, Se, and Sp, respectively.
    Default is uniform prior for all parameters - Beta(1, 1).
    A single vector of the form `c(a, b)` can be provided for each.

min\_mean\_se, min\_mean\_sp, max\_mean\_se, max\_mean\_se
:   Minimum
    and maximum prior mean for sensitivity (se) and specificity (sp).

n\_thresholds
:   Number of thresholds (int.).

## On this page

Developed by Giuliano Netto Flores Cruz.

Site built with pkgdown 2.0.6.

Using preferably template.
