# Supplementary material for: Bayesian Decision Curve Analysis With Bayesdca
Source: Stat Med. 2024 Dec 1;43(30):6042–58. doi: 10.1002/sim.10277 (PMC11639651; doi:10.1002/sim.10277)
Supplement: Supplementary file 1 — Appendix S1. Supplementary methods. [file SIM-43-6042-s001.zip › sim10277-sup-0003-DataS1.zip!/bayesdca-5295e3c320f747cf50839b2aa3ddae936e69f45e/docs/reference/dot-get_prior_parameters.html]

 Get priors for Bayesian DCA — .get\_prior\_parameters • bayesDCA         
Skip to contents


bayesDCA
0.0.0.9000


- Reference
- Articles

  Survival outcomes

# Get priors for Bayesian DCA

Source: `R/utils.R`

`dot-get_prior_parameters.Rd`

Get priors for Bayesian DCA

## Usage

```
.get_prior_parameters(
  thresholds,
  threshold_varying_prior = FALSE,
  n_strategies = NULL,
  prior_p = NULL,
  prior_se = NULL,
  prior_sp = NULL,
  ignorance_region_cutpoints = c(0.25, 0.75) * max(thresholds),
  min_sens_prior_mean = 0.01,
  max_sens_prior_mean = 0.99,
  max_sens_prior_sample_size = 5,
  ignorance_region_mean = 0.5,
  ignorance_region_sample_size = 2,
  prev_prior_mean = 0.5,
  prev_prior_sample_size = 2
)
```

## Arguments

thresholds
:   Vector of decision thresholds.

shift
:   Scalar controlling height of prior
    Specificity curve. Only used if `constant=FALSE`.

slope
:   Scalar controlling shape of prior
    Specificity curve. Only used if `constant=FALSE`.

min\_mean\_se, min\_mean\_sp, max\_mean\_se, max\_mean\_se
:   Minimum

prior\_sample\_size
:   Prior sample size of strength.

min\_prior\_sample\_size
:   Minimum prior sample size or strength.

max\_prior\_sample\_size
:   Maximum prior sample size or strength.

slope\_prior\_sample\_size
:   Rate of change in prior
    sample size or strength.
    and maximum prior mean for sensitivity (se) and specificity (sp).

## On this page

Developed by Giuliano Netto Flores Cruz.

Site built with pkgdown 2.0.7.

Using preferably template.
