# Supplementary material for: Bayesian Decision Curve Analysis With Bayesdca
Source: Stat Med. 2024 Dec 1;43(30):6042–58. doi: 10.1002/sim.10277 (PMC11639651; doi:10.1002/sim.10277)
Supplement: Supplementary file 1 — Appendix S1. Supplementary methods. [file SIM-43-6042-s001.zip › sim10277-sup-0003-DataS1.zip!/bayesdca-5295e3c320f747cf50839b2aa3ddae936e69f45e/docs/reference/dot-get_relevant_pars.html]

 Get relevant parameters to parse from Stan output (BayesDCASurv) — .get\_relevant\_pars • bayesDCA         
Skip to contents


bayesDCA
0.0.0.9000


- Reference
- Articles

  Survival outcomes

# Get relevant parameters to parse from Stan output (BayesDCASurv)

Source: `R/dca_surv.R`

`dot-get_relevant_pars.Rd`

Get relevant parameters to parse from Stan output (BayesDCASurv)

## Usage

```
.get_relevant_pars()
```

Developed by Giuliano Netto Flores Cruz.

Site built with pkgdown 2.0.7.

Using preferably template.
