# Supplementary material for: Bayesian Decision Curve Analysis With Bayesdca
Source: Stat Med. 2024 Dec 1;43(30):6042–58. doi: 10.1002/sim.10277 (PMC11639651; doi:10.1002/sim.10277)
Supplement: Supplementary file 1 — Appendix S1. Supplementary methods. [file SIM-43-6042-s001.zip › sim10277-sup-0003-DataS1.zip!/bayesdca-5295e3c320f747cf50839b2aa3ddae936e69f45e/docs/reference/dot-get_thr_data_list.html]

 Get Threshold Performance Data for DCA list — .get\_thr\_data\_list • bayesDCA         
Skip to contents


bayesDCA
0.0.0.9000


- Reference
- Articles

  Survival outcomes

# Get Threshold Performance Data for DCA list

Source: `R/dca.R`

`dot-get_thr_data_list.Rd`

Get Threshold Performance Data for DCA list

## Usage

```
.get_thr_data_list(
  .data,
  thresholds = seq(0.01, 0.5, 0.01),
  prior_only = FALSE
)
```

## Arguments

.data
:   A data.frame with an `outcomes` column (0 or 1 for each individual)
    and one or more columns with predicted probabilities from each of desired list
    of predictive models, or with 0 or 1 indicator from each of desired list of
    binary tests.

thresholds
:   Decision thresholds.

prior\_only
:   If TRUE, returns prior only (ignores any data).

## On this page

Developed by Giuliano Netto Flores Cruz.

Site built with pkgdown 2.0.7.

Using preferably template.
