# Supplementary material for: Bayesian Decision Curve Analysis With Bayesdca
Source: Stat Med. 2024 Dec 1;43(30):6042–58. doi: 10.1002/sim.10277 (PMC11639651; doi:10.1002/sim.10277)
Supplement: Supplementary file 1 — Appendix S1. Supplementary methods. [file SIM-43-6042-s001.zip › sim10277-sup-0003-DataS1.zip!/bayesdca-5295e3c320f747cf50839b2aa3ddae936e69f45e/docs/reference/dot-get_threshold_varying_prior_parameters.html]

 Get threshold-varying priors for Bayesian DCA — .get\_threshold\_varying\_prior\_parameters • bayesDCA         
Skip to contents


bayesDCA
0.0.0.9000


- Reference
- Articles

  Survival outcomes

# Get threshold-varying priors for Bayesian DCA

Source: `R/utils.R`

`dot-get_threshold_varying_prior_parameters.Rd`

Get threshold-varying priors for Bayesian DCA

## Usage

```
.get_threshold_varying_prior_parameters(
  thresholds,
  n_strategies,
  ignorance_region_cutpoints = c(0.25, 0.75) * max(thresholds),
  min_sens_prior_mean = 0.01,
  max_sens_prior_mean = 0.99,
  max_sens_prior_sample_size = 5,
  ignorance_region_mean = 0.5,
  ignorance_region_sample_size = 2,
  prev_prior_mean = 0.5,
  prev_prior_sample_size = 2
)
```

Developed by Giuliano Netto Flores Cruz.

Site built with pkgdown 2.0.7.

Using preferably template.
