# Supplementary material for: Bayesian Decision Curve Analysis With Bayesdca
Source: Stat Med. 2024 Dec 1;43(30):6042–58. doi: 10.1002/sim.10277 (PMC11639651; doi:10.1002/sim.10277)
Supplement: Supplementary file 1 — Appendix S1. Supplementary methods. [file SIM-43-6042-s001.zip › sim10277-sup-0003-DataS1.zip!/bayesdca-5295e3c320f747cf50839b2aa3ddae936e69f45e/docs/reference/extract_dca_draws.html]

 Get posterior draws from DCA stanfit — extract\_dca\_draws • bayesDCA         
Skip to contents


bayesDCA
0.0.0.9000


- Reference

# Get posterior draws from DCA stanfit

Source: `R/dca.R`

`extract_dca_draws.Rd`

Get posterior draws from DCA stanfit

## Usage

```
extract_dca_draws(fit, model_or_test_names)
```

## Arguments

fit
:   A stanfit object.

model\_or\_test\_names
:   Vector of names of models or binary tests under assessment.

## On this page

Developed by Giuliano Netto Flores Cruz.

Site built with pkgdown 2.0.3.

Using preferably template.
