# Supplementary material for: Bayesian Decision Curve Analysis With Bayesdca
Source: Stat Med. 2024 Dec 1;43(30):6042–58. doi: 10.1002/sim.10277 (PMC11639651; doi:10.1002/sim.10277)
Supplement: Supplementary file 1 — Appendix S1. Supplementary methods. [file SIM-43-6042-s001.zip › sim10277-sup-0003-DataS1.zip!/bayesdca-5295e3c320f747cf50839b2aa3ddae936e69f45e/docs/reference/extract_dca_summary.html]

 Get summary from BayesDCA fit — extract\_dca\_summary • bayesDCA         
Skip to contents


bayesDCA
0.0.0.9000


- Reference

# Get summary from BayesDCA fit

Source: `R/dca.R`

`extract_dca_summary.Rd`

Get summary from BayesDCA fit

## Usage

```
extract_dca_summary(
  fit,
  model_or_test_names,
  summary_probs = c(0.025, 0.975),
  thresholds = seq(0.01, 0.5, 0.01)
)
```

## Arguments

fit
:   A stanfit object.

model\_or\_test\_names
:   Vector of names of models or binary tests under assessment.

summary\_probs
:   Numeric vector giving probabilities for credible interval.

thresholds
:   Vector of thresholds for DCA.

## On this page

Developed by Giuliano Netto Flores Cruz.

Site built with pkgdown 2.0.3.

Using preferably template.
