# Supplementary material for: Bayesian Decision Curve Analysis With Bayesdca
Source: Stat Med. 2024 Dec 1;43(30):6042–58. doi: 10.1002/sim.10277 (PMC11639651; doi:10.1002/sim.10277)
Supplement: Supplementary file 1 — Appendix S1. Supplementary methods. [file SIM-43-6042-s001.zip › sim10277-sup-0003-DataS1.zip!/bayesdca-5295e3c320f747cf50839b2aa3ddae936e69f45e/docs/reference/get_colors_and_labels.html]

 Get colors and labels for BayesDCA plots — get\_colors\_and\_labels • bayesDCA         
Skip to contents


bayesDCA
0.0.0.9000


- Reference
- Articles

  Survival outcomes

# Get colors and labels for BayesDCA plots

Source: `R/utils.R`

`get_colors_and_labels.Rd`

Get colors and labels for BayesDCA plots

## Usage

```
get_colors_and_labels(
  obj,
  strategies = NULL,
  colors = NULL,
  labels = NULL,
  all_or_none = TRUE
)
```

## Arguments

obj
:   BayesDCAList object

colors
:   Named vector with color for each model or test. If provided
    for a subset of models or tests, only that subset will be plotted.

labels
:   Named vector with label for each model or test.

## On this page

Developed by Giuliano Netto Flores Cruz.

Site built with pkgdown 2.0.7.

Using preferably template.
