# Supplementary material for: Bayesian Decision Curve Analysis With Bayesdca
Source: Stat Med. 2024 Dec 1;43(30):6042–58. doi: 10.1002/sim.10277 (PMC11639651; doi:10.1002/sim.10277)
Supplement: Supplementary file 1 — Appendix S1. Supplementary methods. [file SIM-43-6042-s001.zip › sim10277-sup-0003-DataS1.zip!/bayesdca-5295e3c320f747cf50839b2aa3ddae936e69f45e/docs/reference/get_cutpoints.html]

 Get cutpoints for survival estimation — get\_cutpoints • bayesDCA         
Skip to contents


bayesDCA
0.0.0.9000


- Reference
- Articles

  Survival outcomes

# Get cutpoints for survival estimation

Source: `R/utils.R`

`get_cutpoints.Rd`

Get cutpoints for survival estimation

## Usage

```
get_cutpoints(
  .prediction_time,
  .event_times,
  .min_events = 10,
  .base_cutpoints = NULL
)
```

## Arguments

.prediction\_time
:   time point at which event is predicted to happen

.event\_times
:   times of observed events (non-censored)

.base\_cutpoints
:   vector of cutpoints to start with

## On this page

Developed by Giuliano Netto Flores Cruz.

Site built with pkgdown 2.0.7.

Using preferably template.
