# Supplementary material for: Bayesian Decision Curve Analysis With Bayesdca
Source: Stat Med. 2024 Dec 1;43(30):6042–58. doi: 10.1002/sim.10277 (PMC11639651; doi:10.1002/sim.10277)
Supplement: Supplementary file 1 — Appendix S1. Supplementary methods. [file SIM-43-6042-s001.zip › sim10277-sup-0003-DataS1.zip!/bayesdca-5295e3c320f747cf50839b2aa3ddae936e69f45e/docs/reference/get_events_per_interval.html]

 Get events per intervals defined by set of cutpoints — get\_events\_per\_interval • bayesDCA         
Skip to contents


bayesDCA
0.0.0.9000


- Reference
- Articles

  Survival outcomes

# Get events per intervals defined by set of cutpoints

Source: `R/utils.R`

`get_events_per_interval.Rd`

Get events per intervals defined by set of cutpoints

## Usage

```
get_events_per_interval(.cutpoints, .event_times)
```

## Arguments

.cutpoints
:   cutpoints defining intervals

.event\_times
:   times of observed events (non-censored)

## On this page

Developed by Giuliano Netto Flores Cruz.

Site built with pkgdown 2.0.7.

Using preferably template.
