# Supplementary material for: Bayesian Decision Curve Analysis With Bayesdca
Source: Stat Med. 2024 Dec 1;43(30):6042–58. doi: 10.1002/sim.10277 (PMC11639651; doi:10.1002/sim.10277)
Supplement: Supplementary file 1 — Appendix S1. Supplementary methods. [file SIM-43-6042-s001.zip › sim10277-sup-0003-DataS1.zip!/bayesdca-5295e3c320f747cf50839b2aa3ddae936e69f45e/docs/reference/get_positivity_posterior_parameters.html]

 Get posterior parameters for positivity probability — get\_positivity\_posterior\_parameters • bayesDCA         
Skip to contents


bayesDCA
0.0.0.9000


- Reference
- Articles

  Survival outcomes

# Get posterior parameters for positivity probability

Source: `R/utils.R`

`get_positivity_posterior_parameters.Rd`

Get posterior parameters for positivity probability

## Usage

```
get_positivity_posterior_parameters(
  .prediction_data,
  .thresholds,
  .prior_shape1 = 1,
  .prior_shape2 = 1,
  .prior_only = FALSE
)
```

## Arguments

.prediction\_data
:   Contains prognostic model predictions

.thresholds
:   DCA thresholds.

.prior\_shape1
:   Shape 1 for beta prior.

.prior\_shape2
:   Shape 2 for beta prior.

## On this page

Developed by Giuliano Netto Flores Cruz.

Site built with pkgdown 2.0.7.

Using preferably template.
