# Supplementary material for: Bayesian Decision Curve Analysis With Bayesdca
Source: Stat Med. 2024 Dec 1;43(30):6042–58. doi: 10.1002/sim.10277 (PMC11639651; doi:10.1002/sim.10277)
Supplement: Supplementary file 1 — Appendix S1. Supplementary methods. [file SIM-43-6042-s001.zip › sim10277-sup-0003-DataS1.zip!/bayesdca-5295e3c320f747cf50839b2aa3ddae936e69f45e/docs/reference/get_prior_parameters.html]

 Get prior parameters formated for Stan model — get\_prior\_parameters • bayesDCA         
Skip to contents


bayesDCA
0.0.0.9000


- Reference

# Get prior parameters formated for Stan model

Source: `R/dca.R`

`get_prior_parameters.Rd`

Get prior parameters formated for Stan model

## Usage

```
get_prior_parameters(
  n_thresholds,
  n_models_or_tests,
  prior_p = NULL,
  prior_se = NULL,
  prior_sp = NULL
)
```

## Arguments

n\_thresholds
:   Number of thresholds (int.).

n\_models\_or\_tests
:   Number of models or tests (int.).

prior\_p, prior\_se, prior\_sp
:   Non-negative shape values for
    Beta(alpha, beta) priors used for p, Se, and Sp, respectively.
    Default is uniform prior for all parameters - Beta(1, 1).
    A single vector of the form `c(a, b)` can be provided for each.

## On this page

Developed by Giuliano Netto Flores Cruz.

Site built with pkgdown 2.0.3.

Using preferably template.
