# Supplementary material for: Bayesian Decision Curve Analysis With Bayesdca
Source: Stat Med. 2024 Dec 1;43(30):6042–58. doi: 10.1002/sim.10277 (PMC11639651; doi:10.1002/sim.10277)
Supplement: Supplementary file 1 — Appendix S1. Supplementary methods. [file SIM-43-6042-s001.zip › sim10277-sup-0003-DataS1.zip!/bayesdca-5295e3c320f747cf50839b2aa3ddae936e69f45e/docs/reference/get_prior_sample_size.html]

 Get threshold-specific prior sample size or strength — get\_prior\_sample\_size • bayesDCA         
Skip to contents


bayesDCA
0.0.0.9000


- Reference
- Articles

  Survival outcomes

# Get threshold-specific prior sample size or strength

Source: `R/utils.R`

`get_prior_sample_size.Rd`

Get threshold-specific prior sample size or strength

## Usage

```
get_prior_sample_size(
  thresholds,
  min_prior_sample_size = 20,
  max_prior_sample_size = 100,
  slope_prior_sample_size = 300
)
```

## Arguments

thresholds
:   Decision thresholds

min\_prior\_sample\_size
:   Minimum prior sample size or strength.

max\_prior\_sample\_size
:   Maximum prior sample size or strength.

slope\_prior\_sample\_size
:   Rate of change in prior sample size or strength.

## On this page

Developed by Giuliano Netto Flores Cruz.

Site built with pkgdown 2.0.6.

Using preferably template.
