# Supplementary material for: Bayesian Decision Curve Analysis With Bayesdca
Source: Stat Med. 2024 Dec 1;43(30):6042–58. doi: 10.1002/sim.10277 (PMC11639651; doi:10.1002/sim.10277)
Supplement: Supplementary file 1 — Appendix S1. Supplementary methods. [file SIM-43-6042-s001.zip › sim10277-sup-0003-DataS1.zip!/bayesdca-5295e3c320f747cf50839b2aa3ddae936e69f45e/docs/reference/get_prior_se_mu.html]

 Get threshold-specific mean prior sensitivity — get\_prior\_se\_mu • bayesDCA         
Skip to contents


bayesDCA
0.0.0.9000


- Reference
- Articles

  Survival outcomes

# Get threshold-specific mean prior sensitivity

Source: `R/utils.R`

`get_prior_se_mu.Rd`

Get threshold-specific mean prior sensitivity

## Usage

```
get_prior_se_mu(
  thresholds,
  shift = 0.45,
  slope = 0.025,
  .min = 0.1,
  .max = 0.9
)
```

## Arguments

thresholds
:   Vector of decision thresholds.

shift
:   Scalar controlling height of prior Sensitivity curve

slope
:   Scalar controlling shape of prior Sensitivity curve

.min, .max
:   Minimum and maximum prior mean

## On this page

Developed by Giuliano Netto Flores Cruz.

Site built with pkgdown 2.0.6.

Using preferably template.
