# Supplementary material for: Bayesian Decision Curve Analysis With Bayesdca
Source: Stat Med. 2024 Dec 1;43(30):6042–58. doi: 10.1002/sim.10277 (PMC11639651; doi:10.1002/sim.10277)
Supplement: Supplementary file 1 — Appendix S1. Supplementary methods. [file SIM-43-6042-s001.zip › sim10277-sup-0003-DataS1.zip!/bayesdca-5295e3c320f747cf50839b2aa3ddae936e69f45e/docs/reference/get_superiority_prob_plot_data_surv.html]

 Get delta plot data for survival bayesDCA — get\_superiority\_prob\_plot\_data\_surv • bayesDCA         
Skip to contents


bayesDCA
0.0.0.9000


- Reference
- Articles

  Survival outcomes

# Get delta plot data for survival bayesDCA

Source: `R/dca_surv.R`

`get_superiority_prob_plot_data_surv.Rd`

Get delta plot data for survival bayesDCA

## Usage

```
get_superiority_prob_plot_data_surv(
  obj,
  min_diff = 0,
  strategies = NULL,
  type = c("best", "useful", "pairwise"),
  labels = NULL
)
```

## Arguments

obj
:   BayesDCA object

min\_diff
:   Minimum difference to call superior.

strategies
:   Character vector with subset of decision strategies.

type
:   One of "best", "useful", or "pairwise".

labels
:   Named vector with label for each model or test.

## Value

A ggplot object.

## On this page

Developed by Giuliano Netto Flores Cruz.

Site built with pkgdown 2.0.7.

Using preferably template.
