# Supplementary material for: Bayesian Decision Curve Analysis With Bayesdca
Source: Stat Med. 2024 Dec 1;43(30):6042–58. doi: 10.1002/sim.10277 (PMC11639651; doi:10.1002/sim.10277)
Supplement: Supplementary file 1 — Appendix S1. Supplementary methods. [file SIM-43-6042-s001.zip › sim10277-sup-0003-DataS1.zip!/bayesdca-5295e3c320f747cf50839b2aa3ddae936e69f45e/docs/reference/get_survival_posterior_parameters.html]

 Get posterior parameters for survival model — get\_survival\_posterior\_parameters • bayesDCA         
Skip to contents


bayesDCA
0.0.0.9000


- Reference
- Articles

  Survival outcomes

# Get posterior parameters for survival model

Source: `R/utils.R`

`get_survival_posterior_parameters.Rd`

Get posterior parameters for survival model

## Usage

```
get_survival_posterior_parameters(
  .prediction_data,
  .surv_data,
  .cutpoints,
  .models_or_tests,
  .thresholds,
  .prior_scaling_factor,
  .prior_means = NULL
)
```

## Arguments

.prediction\_data
:   Contains prognostic model predictions

.surv\_data
:   Contains observed time to event (`.time` column) and event indicator (`.status` column)

.cutpoints
:   Survival cutpoints.

.models\_or\_tests
:   Names for models or tests.

.thresholds
:   DCA thresholds.

.prior\_scaling\_factor
:   Prior alpha = (0.69/median\_surv)\*.prior\_scaling\_factor, Prior beta = .prior\_scaling\_factor.

## On this page

Developed by Giuliano Netto Flores Cruz.

Site built with pkgdown 2.0.6.

Using preferably template.
