# Supplementary material for: Bayesian Decision Curve Analysis With Bayesdca
Source: Stat Med. 2024 Dec 1;43(30):6042–58. doi: 10.1002/sim.10277 (PMC11639651; doi:10.1002/sim.10277)
Supplement: Supplementary file 1 — Appendix S1. Supplementary methods. [file SIM-43-6042-s001.zip › sim10277-sup-0003-DataS1.zip!/bayesdca-5295e3c320f747cf50839b2aa3ddae936e69f45e/docs/reference/get_survival_time_exposed.html]

 Get time exposed within each interval for a given prediction time — get\_survival\_time\_exposed • bayesDCA         
Skip to contents


bayesDCA
0.0.0.9000


- Reference
- Articles

  Survival outcomes

# Get time exposed within each interval for a given prediction time

Source: `R/utils.R`

`get_survival_time_exposed.Rd`

Get time exposed within each interval for a given prediction time

## Usage

```
get_survival_time_exposed(.prediction_time, .cutpoints)
```

## Arguments

.prediction\_time
:   Time for event prediction

.cutpoints
:   Cutpoints for constant hazard interval

## On this page

Developed by Giuliano Netto Flores Cruz.

Site built with pkgdown 2.0.7.

Using preferably template.
