# Supplementary material for: Bayesian Decision Curve Analysis With Bayesdca
Source: Stat Med. 2024 Dec 1;43(30):6042–58. doi: 10.1002/sim.10277 (PMC11639651; doi:10.1002/sim.10277)
Supplement: Supplementary file 1 — Appendix S1. Supplementary methods. [file SIM-43-6042-s001.zip › sim10277-sup-0003-DataS1.zip!/bayesdca-5295e3c320f747cf50839b2aa3ddae936e69f45e/docs/reference/get_thr_data.html]

 Get Threshold Performance Data — get\_thr\_data • bayesDCA         
Skip to contents


bayesDCA
0.0.0.9000


- Reference
- Articles

  Survival outcomes

# Get Threshold Performance Data

Source: `R/dca.R`

`get_thr_data.Rd`

Get Threshold Performance Data

## Usage

```
get_thr_data(outcomes, predictions, thresholds = seq(0.01, 0.5, 0.01))
```

## Arguments

outcomes
:   Integer vector (0 or 1) with binary outcomes.

predictions
:   Numeric vector with predicted probabilities.

## On this page

Developed by Giuliano Netto Flores Cruz.

Site built with pkgdown 2.0.6.

Using preferably template.
