# Supplementary material for: Bayesian Decision Curve Analysis With Bayesdca
Source: Stat Med. 2024 Dec 1;43(30):6042–58. doi: 10.1002/sim.10277 (PMC11639651; doi:10.1002/sim.10277)
Supplement: Supplementary file 1 — Appendix S1. Supplementary methods. [file SIM-43-6042-s001.zip › sim10277-sup-0003-DataS1.zip!/bayesdca-5295e3c320f747cf50839b2aa3ddae936e69f45e/docs/reference/index.html]

 Function reference • bayesDCA         
Skip to contents


bayesDCA
0.0.0.9000


- Reference
- Articles

  Survival outcomes

# Function reference

## Main

`dca()`
:   Bayesian Decision Curve Analysis for Predictive Models and Binary tests

`dca_surv()`
:   Bayesian Decision Curve Analysis
    for Survival outcomes

`compare_dca()`
:   Plot BayesDCA comparison

## Datasets

`PredModelData`
:   Predictive Model Evaluation Data.

`dca_survival_data`
:   Survival Data for Bayesian DCA.

`Docking2021Data`
:   Survival data from Docking, 2021 - Figures 3B-3E

## Other functions

`plot_delta()`
:   Plot BayesDCA delta

`plot_superiority_prob()`
:   Plot P(useful) from Wynants 2018 (doi: 10.1002/sim.7653)

`plot_evpi()`
:   Plot Expected Value of Perfect Information (EVPI)

`plot_ppc()`
:   Plot prior predictive check for BayesDCA (binary case only)

`plot(<BayesDCA>)`
:   Plot BayesDCA

`plot(<BayesDCASurv>)`
:   Plot BayesDCASurv

`print(<BayesDCA>)`
:   Print BayesDCA

`print(<BayesDCASurv>)`
:   Print BayesDCASurv

`bayesDCA-package` `bayesDCA`
:   The 'bayesDCA' package.

## On this page

Developed by Giuliano Netto Flores Cruz.

Site built with pkgdown 2.0.7.

Using preferably template.
