# Supplementary material for: Bayesian Decision Curve Analysis With Bayesdca
Source: Stat Med. 2024 Dec 1;43(30):6042–58. doi: 10.1002/sim.10277 (PMC11639651; doi:10.1002/sim.10277)
Supplement: Supplementary file 1 — Appendix S1. Supplementary methods. [file SIM-43-6042-s001.zip › sim10277-sup-0003-DataS1.zip!/bayesdca-5295e3c320f747cf50839b2aa3ddae936e69f45e/docs/reference/plot.BayesDCA.html]

 Plot BayesDCA — plot.BayesDCA • bayesDCA         
Skip to contents


bayesDCA
0.0.0.9000


- Reference
- Articles

  Survival outcomes

# Plot BayesDCA

Source: `R/plotting.R`

`plot.BayesDCA.Rd`

Plot BayesDCA

## Usage

```
# S3 method for BayesDCA
plot(
  obj,
  strategies = NULL,
  colors = NULL,
  labels = NULL,
  raw_values = NULL,
  raw_values_label = "Biomarker threshold",
  linewidth = 1.5,
  ...
)
```

## Arguments

obj
:   BayesDCA object

strategies
:   Character vector with models or tests to plot.

colors
:   Named vector with color for each model or test. If provided
    for a subset of models or tests, only that subset will be plotted.

labels
:   Named vector with label for each model or test.

raw\_values
:   Add raw predicted values on x-axis.

raw\_values\_label
:   Label for raw values.

linewidth
:   Width of plotted lines.

## On this page

Developed by Giuliano Netto Flores Cruz.

Site built with pkgdown 2.0.7.

Using preferably template.
