# Supplementary material for: Bayesian Decision Curve Analysis With Bayesdca
Source: Stat Med. 2024 Dec 1;43(30):6042–58. doi: 10.1002/sim.10277 (PMC11639651; doi:10.1002/sim.10277)
Supplement: Supplementary file 1 — Appendix S1. Supplementary methods. [file SIM-43-6042-s001.zip › sim10277-sup-0003-DataS1.zip!/bayesdca-5295e3c320f747cf50839b2aa3ddae936e69f45e/docs/reference/plot_classification.html]

 Plot classification performance from Bayesian DCA of binary outcome — plot\_classification • bayesDCA         
Skip to contents


bayesDCA
0.0.0.9000


- Reference
- Articles

  Survival outcomes

# Plot classification performance from Bayesian DCA of binary outcome

Source: `R/plotting.R`

`plot_classification.Rd`

May plot either sensitivity or specificity.

## Usage

```
plot_classification(
  obj,
  type = c("sensitivity", "specificity"),
  models_or_tests = NULL,
  colors = NULL,
  labels = NULL
)
```

## Arguments

obj
:   BayesDCAList object

models\_or\_tests
:   Character vector with models or tests to compare. If null, compares either first two in `obj$model_or_test_names` or the first one against Treat all/none (if only one available).

colors
:   Named vector with color for each model or test. If provided
    for a subset of models or tests, only that subset will be plotted.

labels
:   Named vector with label for each model or test.

## On this page

Developed by Giuliano Netto Flores Cruz.

Site built with pkgdown 2.0.6.

Using preferably template.
