# Supplementary material for: Bayesian Decision Curve Analysis With Bayesdca
Source: Stat Med. 2024 Dec 1;43(30):6042–58. doi: 10.1002/sim.10277 (PMC11639651; doi:10.1002/sim.10277)
Supplement: Supplementary file 1 — Appendix S1. Supplementary methods. [file SIM-43-6042-s001.zip › sim10277-sup-0003-DataS1.zip!/bayesdca-5295e3c320f747cf50839b2aa3ddae936e69f45e/docs/reference/plot_ppc.html]

 Plot prior predictive check for BayesDCA (binary case only) — plot\_ppc • bayesDCA         
Skip to contents


bayesDCA
0.0.0.9000


- Reference
- Articles

  Survival outcomes

# Plot prior predictive check for BayesDCA (binary case only)

Source: `R/plotting.R`

`plot_ppc.Rd`

Plot prior predictive check for BayesDCA (binary case only)

## Usage

```
plot_ppc(obj, plot_list = FALSE, n_draws = 4000, bins = 20)
```

## Arguments

obj
:   BayesDCA object

plot\_list
:   If TRUE, returns a list with each separate plot.

n\_draws
:   Number of prior draws to use.

bins
:   Number of bins to use in the histogram of prior prevalence.

## Examples

```
df <- data.frame(outcomes = 1, x = 1)  # specific values don't really matter
fit <- dca(df, prior_only = TRUE, threshold_varying_prior = TRUE)
plot_ppc(fit)
```

## On this page

Developed by Giuliano Netto Flores Cruz.

Site built with pkgdown 2.0.7.

Using preferably template.
