# Supplementary material for: Bayesian Decision Curve Analysis With Bayesdca
Source: Stat Med. 2024 Dec 1;43(30):6042–58. doi: 10.1002/sim.10277 (PMC11639651; doi:10.1002/sim.10277)
Supplement: Supplementary file 1 — Appendix S1. Supplementary methods. [file SIM-43-6042-s001.zip › sim10277-sup-0003-DataS1.zip!/bayesdca-5295e3c320f747cf50839b2aa3ddae936e69f45e/docs/reference/plot_prob_better.html]

 Plot probability of better net benefit between two models or tests — plot\_prob\_better • bayesDCA         
Skip to contents


bayesDCA
0.0.0.9000


- Reference

# Plot probability of better net benefit between two models or tests

Source: `R/dca.R`

`plot_prob_better.Rd`

Plot probability of better net benefit between two models or tests

## Usage

```
plot_prob_better(obj, models_or_tests = NULL, labels = NULL)
```

## Arguments

obj
:   BayesDCAList object

models\_or\_tests
:   Character vector with models or tests to compare. If null, compares either first two in `obj$model_or_test_names` or the first one against Treat all/none (if only one available).

labels
:   Named vector with label for each model or test.

colors
:   Named vector with color for each model or test. If provided
    for a subset of models or tests, only that subset will be plotted.

## Value

A ggplot object.

## Examples

```
data(PredModelData)
fit <- dca(PredModelData, cores = 4)
plot_prob_better(fit)
```

## On this page

Developed by Giuliano Netto Flores Cruz.

Site built with pkgdown 2.0.3.

Using preferably template.
