# Supplementary material for: Bayesian Decision Curve Analysis With Bayesdca
Source: Stat Med. 2024 Dec 1;43(30):6042–58. doi: 10.1002/sim.10277 (PMC11639651; doi:10.1002/sim.10277)
Supplement: Supplementary file 1 — Appendix S1. Supplementary methods. [file SIM-43-6042-s001.zip › sim10277-sup-0003-DataS1.zip!/bayesdca-5295e3c320f747cf50839b2aa3ddae936e69f45e/docs/reference/plot_superiority_prob.html]

 Plot P(useful) from Wynants 2018 (doi: 10.1002/sim.7653) — plot\_superiority\_prob • bayesDCA         
Skip to contents


bayesDCA
0.0.0.9000


- Reference
- Articles

  Survival outcomes

# Plot P(useful) from Wynants 2018 (doi: 10.1002/sim.7653)

Source: `R/plotting.R`

`plot_superiority_prob.Rd`

Plot P(useful) from Wynants 2018 (doi: 10.1002/sim.7653)

## Usage

```
plot_superiority_prob(
  obj,
  strategies = NULL,
  type = c("best", "useful", "pairwise"),
  min_diff = 0,
  colors = NULL,
  labels = NULL,
  linewidth = 1.5
)
```

## Arguments

obj
:   BayesDCA or BayesDCASurv object

strategies
:   Character vector with models or tests to plot. If null, compares either first two in `obj$strategies` or the first one against Treat all/none (if only one available).

type
:   One of "best", "useful", or "pairwise".

min\_diff
:   Minimal difference for superiority. Defaults to zero. Used only for `type = "pairwise"`

colors
:   Named vector with color for each model or test. If provided
    for a subset of models or tests, only that subset will be plotted.

labels
:   Named vector with label for each model or test.

linewidth
:   Width of plotted lines.

plot\_list
:   If TRUE, returns a list of separate ggplot objects.

## Value

A ggplot object.

## Examples

```
data(PredModelData)
fit <- dca(PredModelData)
plot_superiority_prob(fit)
```

## On this page

Developed by Giuliano Netto Flores Cruz.

Site built with pkgdown 2.0.7.

Using preferably template.
