# Supplementary material for: Bayesian Decision Curve Analysis With Bayesdca
Source: Stat Med. 2024 Dec 1;43(30):6042–58. doi: 10.1002/sim.10277 (PMC11639651; doi:10.1002/sim.10277)
Supplement: Supplementary file 1 — Appendix S1. Supplementary methods. [file SIM-43-6042-s001.zip › sim10277-sup-0003-DataS1.zip!/bayesdca-5295e3c320f747cf50839b2aa3ddae936e69f45e/docs/reference/simulate_diagnostic_test_data.html]

 Simulate Diagnostic Test Data — simulate\_diagnostic\_test\_data • bayesDCA         
Skip to contents


bayesDCA
0.0.0.9000


- Reference
- Articles

  Survival outcomes

# Simulate Diagnostic Test Data

Source: `R/fake_data_simulation.R`

`simulate_diagnostic_test_data.Rd`

Simulate Diagnostic Test Data

## Usage

```
simulate_diagnostic_test_data(
  B = 100,
  N = 500,
  true_p = 0.2,
  true_se = 0.9,
  true_sp = 0.9,
  keep_true_pars = FALSE
)
```

## Arguments

B
:   Integer indicating number of simulated datasets.

N
:   Integer indicating sample size in each dataset.

true\_p
:   Proportion indicating true prevalence in each dataset.

true\_se
:   Proportion indicating true sensitivity in each dataset.

true\_sp
:   Proportion indicating true specificity in each dataset.

keep\_true\_pars
:   Logical indicating whether to keep true
    parameter values in the output dataframe.

## Value

A Bx7 data frame with simulated data and provided parameters.

## Details

Diagnostic test data is simulated according to the following
mechanism: for each of the `B` simulations, `d` *diseased* persons
are sampled from a binomial of size `N` and parameter `true_p`. Then,
`tp` *true positive* outcomes are sampled from a binomial of size `d`
and parameter `true_se`, and `tn` *true negative* outcomes are
sampled from a binomial of size `N-d` with parameter `true_sp`. This
simulates `B` diagnostic accuracy studies in which a number of
diseased and non-diseased persons are sampled from a population;
the expected proportion of diseased persons correctly detected as
such is the sensitivity, and the number of non-diseased persons
correctly detected as such is the specificity.

## Examples

```
d <- simulate_diagnostic_test_data(B = 2,
                                   N = 1000,
                                   true_p = 0.2,
                                   true_se = 0.9,
                                   true_sp = 0.9)
#> Error in simulate_diagnostic_test_data(B = 2, N = 1000, true_p = 0.2,     true_se = 0.9, true_sp = 0.9): could not find function "simulate_diagnostic_test_data"
head(d)
#> $summary
#> $net_benefit
#> # A tibble: 41 × 9
#>    par_name   threshold model_or_test_name estimate    sd   `2.5%` `97.5%` n_eff
#>    <chr>          <dbl> <chr>                 <dbl> <dbl>    <dbl>   <dbl> <dbl>
#>  1 net_benef…     0     binary_test           0.471 0.274  0.0241    0.956 7405.
#>  2 net_benef…     0.025 binary_test           0.407 0.256  0.00466   0.894 6957.
#>  3 net_benef…     0.05  binary_test           0.357 0.244 -0.0107    0.841 6968.
#>  4 net_benef…     0.075 binary_test           0.328 0.235 -0.0242    0.807 6529.
#>  5 net_benef…     0.1   binary_test           0.309 0.233 -0.0383    0.794 6533.
#>  6 net_benef…     0.125 binary_test           0.296 0.233 -0.0492    0.783 6702.
#>  7 net_benef…     0.15  binary_test           0.278 0.230 -0.0713    0.752 6489.
#>  8 net_benef…     0.175 binary_test           0.273 0.234 -0.0862    0.763 6050.
#>  9 net_benef…     0.2   binary_test           0.261 0.236 -0.104     0.762 6630.
#> 10 net_benef…     0.225 binary_test           0.249 0.237 -0.125     0.739 6249.
#> # ℹ 31 more rows
#> # ℹ 1 more variable: Rhat <dbl>
#> 
#> $treat_all
#> # A tibble: 41 × 8
#>    par_name      threshold estimate    sd    `2.5%` `97.5%` n_eff  Rhat
#>    <chr>             <dbl>    <dbl> <dbl>     <dbl>   <dbl> <dbl> <dbl>
#>  1 treat_all[1]      0        0.497 0.283  0.0259     0.972 7657.  1.00
#>  2 treat_all[2]      0.025    0.484 0.290  0.000879   0.971 7657.  1.00
#>  3 treat_all[3]      0.05     0.470 0.298 -0.0254     0.971 7657.  1.00
#>  4 treat_all[4]      0.075    0.456 0.306 -0.0531     0.970 7657.  1.00
#>  5 treat_all[5]      0.1      0.441 0.314 -0.0824     0.969 7657.  1.00
#>  6 treat_all[6]      0.125    0.425 0.323 -0.113      0.968 7657.  1.00
#>  7 treat_all[7]      0.15     0.408 0.333 -0.146      0.967 7657.  1.00
#>  8 treat_all[8]      0.175    0.390 0.343 -0.181      0.966 7657.  1.00
#>  9 treat_all[9]      0.2      0.371 0.354 -0.218      0.965 7657.  1.00
#> 10 treat_all[10]     0.225    0.351 0.365 -0.257      0.964 7657.  1.00
#> # ℹ 31 more rows
#> 
#> $prevalence
#> # A tibble: 1 × 7
#>   par_name estimate    sd `2.5%` `97.5%` n_eff  Rhat
#>   <chr>       <dbl> <dbl>  <dbl>   <dbl> <dbl> <dbl>
#> 1 p           0.497 0.283 0.0259   0.972 7657.  1.00
#> 
#> $sensitivity
#> # A tibble: 41 × 9
#>    par_name threshold model_or_test_name estimate     sd `2.5%` `97.5%` n_eff
#>    <chr>        <dbl> <chr>                 <dbl>  <dbl>  <dbl>   <dbl> <dbl>
#>  1 Se[1,1]      0     binary_test           0.948 0.0926  0.672   1.00  5363.
#>  2 Se[2,1]      0.025 binary_test           0.841 0.152   0.448   0.998 7154.
#>  3 Se[3,1]      0.05  binary_test           0.745 0.180   0.328   0.984 7474.
#>  4 Se[4,1]      0.075 binary_test           0.700 0.187   0.287   0.974 7290.
#>  5 Se[5,1]      0.1   binary_test           0.673 0.190   0.253   0.960 8203.
#>  6 Se[6,1]      0.125 binary_test           0.657 0.196   0.240   0.959 6891.
#>  7 Se[7,1]      0.15  binary_test           0.639 0.195   0.215   0.950 9212.
#>  8 Se[8,1]      0.175 binary_test           0.634 0.195   0.223   0.946 6845.
#>  9 Se[9,1]      0.2   binary_test           0.626 0.196   0.209   0.946 8515.
#> 10 Se[10,1]     0.225 binary_test           0.620 0.196   0.204   0.939 6668.
#> # ℹ 31 more rows
#> # ℹ 1 more variable: Rhat <dbl>
#> 
#> $specificity
#> # A tibble: 41 × 9
#>    par_name threshold model_or_test_name estimate     sd    `2.5%` `97.5%` n_eff
#>    <chr>        <dbl> <chr>                 <dbl>  <dbl>     <dbl>   <dbl> <dbl>
#>  1 Sp[1,1]      0     binary_test          0.0477 0.0852   3.68e-8   0.306 5598.
#>  2 Sp[2,1]      0.025 binary_test          0.316  0.188    3.85e-2   0.720 7895.
#>  3 Sp[3,1]      0.05  binary_test          0.453  0.208    8.61e-2   0.863 6999.
#>  4 Sp[4,1]      0.075 binary_test          0.512  0.203    1.36e-1   0.879 6196.
#>  5 Sp[5,1]      0.1   binary_test          0.546  0.204    1.49e-1   0.904 8932.
#>  6 Sp[6,1]      0.125 binary_test          0.567  0.200    1.77e-1   0.913 6294.
#>  7 Sp[7,1]      0.15  binary_test          0.579  0.201    1.78e-1   0.918 9230.
#>  8 Sp[8,1]      0.175 binary_test          0.585  0.204    1.81e-1   0.924 7797.
#>  9 Sp[9,1]      0.2   binary_test          0.594  0.198    1.90e-1   0.924 7120.
#> 10 Sp[10,1]     0.225 binary_test          0.599  0.204    1.85e-1   0.935 7813.
#> # ℹ 31 more rows
#> # ℹ 1 more variable: Rhat <dbl>
#> 
#> attr(,"class")
#> [1] "BayesDCASummary"
#> 
#> $thresholds
#>  [1] 0.000 0.025 0.050 0.075 0.100 0.125 0.150 0.175 0.200 0.225 0.250 0.275
#> [13] 0.300 0.325 0.350 0.375 0.400 0.425 0.450 0.475 0.500 0.525 0.550 0.575
#> [25] 0.600 0.625 0.650 0.675 0.700 0.725 0.750 0.775 0.800 0.825 0.850 0.875
#> [37] 0.900 0.925 0.950 0.975 1.000
#> 
#> $.data
#>     outcomes binary_test
#> 1          0           0
#> 2          0           0
#> 3          0           0
#> 4          0           0
#> 5          0           0
#> 6          0           0
#> 7          0           1
#> 8          0           0
#> 9          0           0
#> 10         0           0
#> 11         0           0
#> 12         0           0
#> 13         0           0
#> 14         1           1
#> 15         0           0
#> 16         0           0
#> 17         0           0
#> 18         0           0
#> 19         0           1
#> 20         1           1
#> 21         0           0
#> 22         1           1
#> 23         0           0
#> 24         0           0
#> 25         0           0
#> 26         0           0
#> 27         0           0
#> 28         1           0
#> 29         0           0
#> 30         0           0
#> 31         0           0
#> 32         0           0
#> 33         0           0
#> 34         0           0
#> 35         0           1
#> 36         0           0
#> 37         0           0
#> 38         0           1
#> 39         0           1
#> 40         0           0
#> 41         1           1
#> 42         0           0
#> 43         0           0
#> 44         0           0
#> 45         0           0
#> 46         0           1
#> 47         0           0
#> 48         0           0
#> 49         0           0
#> 50         0           1
#> 51         0           0
#> 52         0           1
#> 53         0           0
#> 54         0           0
#> 55         0           0
#> 56         0           0
#> 57         0           0
#> 58         0           1
#> 59         0           0
#> 60         0           0
#> 61         0           0
#> 62         0           0
#> 63         0           0
#> 64         0           1
#> 65         1           1
#> 66         0           1
#> 67         0           0
#> 68         0           0
#> 69         0           0
#> 70         0           0
#> 71         1           1
#> 72         0           1
#> 73         1           1
#> 74         0           0
#> 75         0           0
#> 76         0           0
#> 77         0           0
#> 78         0           0
#> 79         1           1
#> 80         0           0
#> 81         0           0
#> 82         0           0
#> 83         0           0
#> 84         0           0
#> 85         1           0
#> 86         0           0
#> 87         0           1
#> 88         0           1
#> 89         1           0
#> 90         0           0
#> 91         0           0
#> 92         0           0
#> 93         0           1
#> 94         0           1
#> 95         0           0
#> 96         0           1
#> 97         0           0
#> 98         0           1
#> 99         0           0
#> 100        0           0
#> 101        0           0
#> 102        0           1
#> 103        0           0
#> 104        0           0
#> 105        0           0
#> 106        0           0
#> 107        0           0
#> 108        0           0
#> 109        0           0
#> 110        0           0
#> 111        0           1
#> 112        0           0
#> 113        0           0
#> 114        0           0
#> 115        0           0
#> 116        0           0
#> 117        0           0
#> 118        0           0
#> 119        0           1
#> 120        0           0
#> 121        0           1
#> 122        0           0
#> 123        0           1
#> 124        0           0
#> 125        0           0
#> 126        0           0
#> 127        0           0
#> 128        0           0
#> 129        0           1
#> 130        0           0
#> 131        0           0
#> 132        0           0
#> 133        0           0
#> 134        0           0
#> 135        0           0
#> 136        0           0
#> 137        0           0
#> 138        0           0
#> 139        0           0
#> 140        1           1
#> 141        0           0
#> 142        0           0
#> 143        1           0
#> 144        0           0
#> 145        0           0
#> 146        0           1
#> 147        0           0
#> 148        1           1
#> 149        0           0
#> 150        0           0
#> 151        1           1
#> 152        0           0
#> 153        0           0
#> 154        0           0
#> 155        0           0
#> 156        0           0
#> 157        0           0
#> 158        0           1
#> 159        0           0
#> 160        0           0
#> 161        0           0
#> 162        0           0
#> 163        0           0
#> 164        1           0
#> 165        0           0
#> 166        0           0
#> 167        0           0
#> 168        0           0
#> 169        0           1
#> 170        1           0
#> 171        0           0
#> 172        0           0
#> 173        0           0
#> 174        0           0
#> 175        0           0
#> 176        0           0
#> 177        0           0
#> 178        0           0
#> 179        0           0
#> 180        0           0
#> 181        0           0
#> 182        1           1
#> 183        0           0
#> 184        0           0
#> 185        0           0
#> 186        0           1
#> 187        0           0
#> 188        0           0
#> 189        0           0
#> 190        0           0
#> 191        0           0
#> 192        0           1
#> 193        0           1
#> 194        0           1
#> 195        1           1
#> 196        0           0
#> 197        0           1
#> 198        0           0
#> 199        1           1
#> 200        0           0
#> 201        0           0
#> 202        0           0
#> 203        0           0
#> 204        0           0
#> 205        0           1
#> 206        0           1
#> 207        0           0
#> 208        0           0
#> 209        0           0
#> 210        0           0
#> 211        0           0
#> 212        0           1
#> 213        1           1
#> 214        0           0
#> 215        1           1
#> 216        0           0
#> 217        0           0
#> 218        1           1
#> 219        1           0
#> 220        0           1
#> 221        0           0
#> 222        0           0
#> 223        1           1
#> 224        1           1
#> 225        0           1
#> 226        0           0
#> 227        0           0
#> 228        0           0
#> 229        0           1
#> 230        0           0
#> 231        0           0
#> 232        0           0
#> 233        0           1
#> 234        0           1
#> 235        0           0
#> 236        0           1
#> 237        0           0
#> 238        1           1
#> 239        0           0
#> 240        0           0
#> 241        0           1
#> 242        0           1
#> 243        0           1
#> 244        0           0
#> 245        0           0
#> 246        0           0
#> 247        0           0
#> 248        1           1
#> 249        0           0
#> 250        0           0
#> 251        0           0
#> 252        0           0
#> 253        0           0
#> 254        0           0
#> 255        0           0
#> 256        0           1
#> 257        0           0
#> 258        0           0
#> 259        0           0
#> 260        0           0
#> 261        1           1
#> 262        0           0
#> 263        0           0
#> 264        0           1
#> 265        0           0
#> 266        0           0
#> 267        0           1
#> 268        0           0
#> 269        0           0
#> 270        0           0
#> 271        0           0
#> 272        0           0
#> 273        1           1
#> 274        0           1
#> 275        0           1
#> 276        0           1
#> 277        0           1
#> 278        0           0
#> 279        0           0
#> 280        0           0
#> 281        0           0
#> 282        0           0
#> 283        1           1
#> 284        1           1
#> 285        0           0
#> 286        1           1
#> 287        0           0
#> 288        0           0
#> 289        0           0
#> 290        0           0
#> 291        0           0
#> 292        0           0
#> 293        0           0
#> 294        0           0
#> 295        1           1
#> 296        1           1
#> 297        0           0
#> 298        0           1
#> 299        0           0
#> 300        0           0
#> 301        0           0
#> 302        0           0
#> 303        0           0
#> 304        0           0
#> 305        1           1
#> 306        0           1
#> 307        0           0
#> 308        0           0
#> 309        0           0
#> 310        1           1
#> 311        0           0
#> 312        0           0
#> 313        0           1
#> 314        0           1
#> 315        0           0
#> 316        0           0
#> 317        0           0
#> 318        1           0
#> 319        0           1
#> 320        0           0
#> 321        0           1
#> 322        1           1
#> 323        0           0
#> 324        0           0
#> 325        0           1
#> 326        0           0
#> 327        0           1
#> 328        0           0
#> 329        0           0
#> 330        0           1
#> 331        1           1
#> 332        1           1
#> 333        0           0
#> 334        0           0
#> 335        0           0
#> 336        0           0
#> 337        0           0
#> 338        0           0
#> 339        0           1
#> 340        0           1
#> 341        0           0
#> 342        0           0
#> 343        0           0
#> 344        0           0
#> 345        0           0
#> 346        0           0
#> 347        1           0
#> 348        0           0
#> 349        0           0
#> 350        0           0
#> 351        0           1
#> 352        0           0
#> 353        0           0
#> 354        0           0
#> 355        0           0
#> 356        0           0
#> 357        1           1
#> 358        0           0
#> 359        0           1
#> 360        0           0
#> 361        1           0
#> 362        1           1
#> 363        0           0
#> 364        0           1
#> 365        1           0
#> 366        0           0
#> 367        0           0
#> 368        0           1
#> 369        0           0
#> 370        0           0
#> 371        0           0
#> 372        0           0
#> 373        0           0
#> 374        0           0
#> 375        1           1
#> 376        0           0
#> 377        1           1
#> 378        0           1
#> 379        0           0
#> 380        0           0
#> 381        0           0
#> 382        0           0
#> 383        1           0
#> 384        0           0
#> 385        0           0
#> 386        0           0
#> 387        0           1
#> 388        0           0
#> 389        0           1
#> 390        0           0
#> 391        1           1
#> 392        0           0
#> 393        0           0
#> 394        0           0
#> 395        0           0
#> 396        0           0
#> 397        0           0
#> 398        0           0
#> 399        1           1
#> 400        0           1
#> 401        0           0
#> 402        0           0
#> 403        0           1
#> 404        0           0
#> 405        0           1
#> 406        1           1
#> 407        0           0
#> 408        0           0
#> 409        0           0
#> 410        0           0
#> 411        0           0
#> 412        0           0
#> 413        0           0
#> 414        0           1
#> 415        0           0
#> 416        0           0
#> 417        0           0
#> 418        0           0
#> 419        0           1
#> 420        0           0
#> 421        0           0
#> 422        0           0
#> 423        1           1
#> 424        1           1
#> 425        0           0
#> 426        0           0
#> 427        0           1
#> 428        0           0
#> 429        0           0
#> 430        0           0
#> 431        0           1
#> 432        0           1
#> 433        0           0
#> 434        0           0
#> 435        0           0
#> 436        0           0
#> 437        0           0
#> 438        0           0
#> 439        0           0
#> 440        0           0
#> 441        0           0
#> 442        0           0
#> 443        0           0
#> 444        0           0
#> 445        1           1
#> 446        0           0
#> 447        0           0
#> 448        0           0
#> 449        0           0
#> 450        0           0
#> 451        0           0
#> 452        0           1
#> 453        0           0
#> 454        0           0
#> 455        0           0
#> 456        0           0
#> 457        1           1
#> 458        0           0
#> 459        0           0
#> 460        0           1
#> 461        0           0
#> 462        1           1
#> 463        0           0
#> 464        0           0
#> 465        0           1
#> 466        1           0
#> 467        0           0
#> 468        0           0
#> 469        0           0
#> 470        0           1
#> 471        0           0
#> 472        0           1
#> 473        0           0
#> 474        0           0
#> 475        0           0
#> 476        0           0
#> 477        0           0
#> 478        0           1
#> 479        0           1
#> 480        1           1
#> 481        0           1
#> 482        0           1
#> 483        0           1
#> 484        0           0
#> 485        0           0
#> 486        0           0
#> 487        0           0
#> 488        0           0
#> 489        0           0
#> 490        0           0
#> 491        0           0
#> 492        0           0
#> 493        1           1
#> 494        0           0
#> 495        0           0
#> 496        0           0
#> 497        0           0
#> 498        0           0
#> 499        0           0
#> 500        0           0
#> 
#> $threshold_data
#> # A tibble: 41 × 6
#>    model_or_test     N     d    tp    tn thresholds
#>    <chr>         <dbl> <dbl> <dbl> <dbl>      <dbl>
#>  1 binary_test       0     0     0     0      0    
#>  2 binary_test       0     0     0     0      0.025
#>  3 binary_test       0     0     0     0      0.05 
#>  4 binary_test       0     0     0     0      0.075
#>  5 binary_test       0     0     0     0      0.1  
#>  6 binary_test       0     0     0     0      0.125
#>  7 binary_test       0     0     0     0      0.15 
#>  8 binary_test       0     0     0     0      0.175
#>  9 binary_test       0     0     0     0      0.2  
#> 10 binary_test       0     0     0     0      0.225
#> # ℹ 31 more rows
#> 
#> $priors
#> $priors$p1
#> [1] 1
#> 
#> $priors$p2
#> [1] 1
#> 
#> $priors$Se1
#>           [,1]
#>  [1,] 4.750000
#>  [2,] 4.214045
#>  [3,] 3.740200
#>  [4,] 3.499080
#>  [5,] 3.357739
#>  [6,] 3.267215
#>  [7,] 3.205887
#>  [8,] 3.162788
#>  [9,] 3.131768
#> [10,] 3.109115
#> [11,] 3.092456
#> [12,] 3.080201
#> [13,] 3.071244
#> [14,] 3.064787
#> [15,] 3.060242
#> [16,] 3.057156
#> [17,] 3.055177
#> [18,] 3.054017
#> [19,] 3.053436
#> [20,] 3.053226
#> [21,] 3.053196
#> [22,] 3.053166
#> [23,] 3.052956
#> [24,] 3.052375
#> [25,] 3.051215
#> [26,] 3.049233
#> [27,] 3.046141
#> [28,] 3.041580
#> [29,] 3.035088
#> [30,] 3.026055
#> [31,] 3.013647
#> [32,] 2.996689
#> [33,] 2.973458
#> [34,] 2.941322
#> [35,] 2.896038
#> [36,] 2.830298
#> [37,] 2.730353
#> [38,] 2.566957
#> [39,] 2.265459
#> [40,] 1.572376
#> [41,] 0.250000
#> 
#> $priors$Se2
#>            [,1]
#>  [1,] 0.2500000
#>  [2,] 0.7859547
#>  [3,] 1.2597995
#>  [4,] 1.5009201
#>  [5,] 1.6422611
#>  [6,] 1.7327851
#>  [7,] 1.7941125
#>  [8,] 1.8372122
#>  [9,] 1.8682323
#> [10,] 1.8908849
#> [11,] 1.9075438
#> [12,] 1.9197988
#> [13,] 1.9287563
#> [14,] 1.9352127
#> [15,] 1.9397583
#> [16,] 1.9428437
#> [17,] 1.9448231
#> [18,] 1.9459830
#> [19,] 1.9465637
#> [20,] 1.9467740
#> [21,] 1.9468038
#> [22,] 1.9468336
#> [23,] 1.9470440
#> [24,] 1.9476248
#> [25,] 1.9487853
#> [26,] 1.9507668
#> [27,] 1.9538586
#> [28,] 1.9584200
#> [29,] 1.9649122
#> [30,] 1.9739453
#> [31,] 1.9863529
#> [32,] 2.0033109
#> [33,] 2.0265415
#> [34,] 2.0586776
#> [35,] 2.1039618
#> [36,] 2.1697021
#> [37,] 2.2696475
#> [38,] 2.4330431
#> [39,] 2.7345415
#> [40,] 3.4276237
#> [41,] 4.7500000
#> 
#> $priors$Sp1
#>           [,1]
#>  [1,] 0.250000
#>  [2,] 1.572376
#>  [3,] 2.265459
#>  [4,] 2.566957
#>  [5,] 2.730353
#>  [6,] 2.830298
#>  [7,] 2.896038
#>  [8,] 2.941322
#>  [9,] 2.973458
#> [10,] 2.996689
#> [11,] 3.013647
#> [12,] 3.026055
#> [13,] 3.035088
#> [14,] 3.041580
#> [15,] 3.046141
#> [16,] 3.049233
#> [17,] 3.051215
#> [18,] 3.052375
#> [19,] 3.052956
#> [20,] 3.053166
#> [21,] 3.053196
#> [22,] 3.053226
#> [23,] 3.053436
#> [24,] 3.054017
#> [25,] 3.055177
#> [26,] 3.057156
#> [27,] 3.060242
#> [28,] 3.064787
#> [29,] 3.071244
#> [30,] 3.080201
#> [31,] 3.092456
#> [32,] 3.109115
#> [33,] 3.131768
#> [34,] 3.162788
#> [35,] 3.205887
#> [36,] 3.267215
#> [37,] 3.357739
#> [38,] 3.499080
#> [39,] 3.740200
#> [40,] 4.214045
#> [41,] 4.750000
#> 
#> $priors$Sp2
#>            [,1]
#>  [1,] 4.7500000
#>  [2,] 3.4276237
#>  [3,] 2.7345415
#>  [4,] 2.4330431
#>  [5,] 2.2696475
#>  [6,] 2.1697021
#>  [7,] 2.1039618
#>  [8,] 2.0586776
#>  [9,] 2.0265415
#> [10,] 2.0033109
#> [11,] 1.9863529
#> [12,] 1.9739453
#> [13,] 1.9649122
#> [14,] 1.9584200
#> [15,] 1.9538586
#> [16,] 1.9507668
#> [17,] 1.9487853
#> [18,] 1.9476248
#> [19,] 1.9470440
#> [20,] 1.9468336
#> [21,] 1.9468038
#> [22,] 1.9467740
#> [23,] 1.9465637
#> [24,] 1.9459830
#> [25,] 1.9448231
#> [26,] 1.9428437
#> [27,] 1.9397583
#> [28,] 1.9352127
#> [29,] 1.9287563
#> [30,] 1.9197988
#> [31,] 1.9075438
#> [32,] 1.8908849
#> [33,] 1.8682323
#> [34,] 1.8372122
#> [35,] 1.7941125
#> [36,] 1.7327851
#> [37,] 1.6422611
#> [38,] 1.5009201
#> [39,] 1.2597995
#> [40,] 0.7859547
#> [41,] 0.2500000
#> 
#> 
#> $model_or_test_names
#> [1] "binary_test"
#>
```

## On this page

Developed by Giuliano Netto Flores Cruz.

Site built with pkgdown 2.0.7.

Using preferably template.
