# Supplementary material for: Bayesian Decision Curve Analysis With Bayesdca
Source: Stat Med. 2024 Dec 1;43(30):6042–58. doi: 10.1002/sim.10277 (PMC11639651; doi:10.1002/sim.10277)
Supplement: Supplementary file 1 — Appendix S1. Supplementary methods. [file SIM-43-6042-s001.zip › sim10277-sup-0003-DataS1.zip!/bayesdca-5295e3c320f747cf50839b2aa3ddae936e69f45e/docs/reference/simulate_prognostic_model_data.html]

 Simulate Prognostic Test Data — simulate\_prognostic\_model\_data • bayesDCA         
Skip to contents


bayesDCA
0.0.0.9000


- Reference
- Articles

  Survival outcomes

# Simulate Prognostic Test Data

Source: `R/fake_data_simulation.R`

`simulate_prognostic_model_data.Rd`

Simulate Prognostic Test Data

## Usage

```
simulate_prognostic_model_data(N = 200, .seed = 123)
```

Developed by Giuliano Netto Flores Cruz.

Site built with pkgdown 2.0.7.

Using preferably template.
