# Supplementary material for: Bayesian Decision Curve Analysis With Bayesdca
Source: Stat Med. 2024 Dec 1;43(30):6042–58. doi: 10.1002/sim.10277 (PMC11639651; doi:10.1002/sim.10277)
Supplement: Supplementary file 1 — Appendix S1. Supplementary methods. [file SIM-43-6042-s001.zip › sim10277-sup-0004-DataS2.zip!/paper-bayesdca-2/extra/sim7653-sup-0001-supporting web material stat med.docx]

**Web appendix 1: WinBUGS code for the weak realistic priors**

model{

for (i in 1:N){

n[i]<-ncases[i]+ncontrols[i]

ncases[i] ~ dbin(prev[i],n[i])

tp[i] ~ dbin(sens[i],ncases[i])

tn[i] ~ dbin(spec[i],ncontrols[i])

logitp[i] ~ dnorm(etap,precp)

logitsens[i] ~ dnorm(etasens[i], precsens)

etasens[i]<-lambdasens0+lambdasens1*logitp[i]

logitspec[i] ~dnorm(etaspec[i], precspec)

etaspec[i]<-lambdaspec0+lambdaspec1*logitp[i]+lambdaspec2*logitsens[i]

prev[i] <- exp(logitp[i])/(1+exp(logitp[i]))

sens[i] <- exp(logitsens[i])/(1+exp(logitsens[i]))

spec[i] <- exp(logitspec[i])/(1+exp(logitspec[i]))

}

#compute summary statistics

mean.p<-etap

mean.sens<-lambdasens0+lambdasens1*etap

mean.spec<-lambdaspec0+lambdaspec1*etap+lambdaspec2*mean.sens

pooledprev <- exp(mean.p)/(1+exp(mean.p))

pooledsens <- exp(mean.sens)/(1+exp(mean.sens))

pooledspec <- exp(mean.spec)/(1+exp(mean.spec))

t<-0.2

pooledNB<-pooledsens*pooledprev-(1-pooledspec)*(1-pooledprev)*t/(1-t)

pooledNB_TA<-pooledprev-(1-pooledprev)*t/(1-t)

pooledNB_p<-pooledsens*.5-(1-pooledspec)*(1-.5)*t/(1-t)

pooledNB_TA_p<-.5-(1-.5)*t/(1-t)

\# priors

etap~dnorm(0,0.001)

lambdasens0~dnorm(0,0.001)

lambdaspec0~dnorm(0, 0.001)

#Fisher or Uniform priors for correlations

zss~dnorm(-0.2, 4)

corr.sens.spec<-(exp(2*zss)-1)/(exp(2*zss)+1)

zsp~dnorm(-0.2, 4)

corr.spec.prev<-(exp(2*zsp)-1)/(exp(2*zsp)+1)

corr.sens.prev~ dunif(-0.99, 0.99)

#Halfnormal priors for between-setting heterogeneity

varprev~dnorm(0, 0.25)I(0, )

varsens~dnorm(0, 0.25)I(0, )

varspec~dnorm(0, 0.25)I(0, )

#Implied relations

precp<-1/varprev

varsens.c<-varsens-pow(lambdasens1,2)*varprev

precsens<-1/varsens.c

varspec.c<-varspec-pow(lambdaspec1,2)*varspec-pow(lambdaspec2,2)*varsens

precspec<-1/varspec.c

lambdasens1<-corr.sens.prev*sqrt(varsens)/sqrt(varprev)

lambdaspec1<-corr.spec.prev*sqrt(varspec)/sqrt(varprev)-lambdaspec2*lambdasens1

lambdaspec2<-(corr.sens.spec*sqrt(varsens)*sqrt(varspec)-corr.spec.prev*sqrt(varprev)*sqrt(varspec)*lambdasens1)/(varsens-pow(lambdasens1,2)*varprev)

\# Predict new triade of sens and spec and prev

logitp.new~ dnorm(etap,precp)

logitsens.new~ dnorm(etasens.new, precsens)

etasens.new<-lambdasens0+lambdasens1*logitp.new

logitspec.new~dnorm(etaspec.new, precspec)

etaspec.new<-lambdaspec0+lambdaspec1*logitp.new+lambdaspec2*logitsens.new

prevnew<-exp(logitp.new)/(1+exp(logitp.new))

sensnew<-exp(logitsens.new)/(1+exp(logitsens.new))

specnew<-exp(logitspec.new)/(1+exp(logitspec.new))

NBnew<-sensnew*prevnew-(1-specnew)*(1-prevnew)*t/(1-t)

NBnew_TA<-prevnew-(1-prevnew)*t/(1-t)

logitp.new.p<-logit(0.5)

logitsens.new.p~ dnorm(etasens.new.p, precsens)

etasens.new.p<-lambdasens0+lambdasens1*logitp.new.p

logitspec.new.p~dnorm(etaspec.new.p, precspec)

etaspec.new.p<-lambdaspec0+lambdaspec1*logitp.new.p+lambdaspec2*logitsens.new.p

sensnew.p<-exp(logitsens.new.p)/(1+exp(logitsens.new.p))

specnew.p<-exp(logitspec.new.p)/(1+exp(logitspec.new.p))

NBnew_p<-sensnew.p*.5-(1-specnew.p)*(1-.5)*t/(1-t)

NBnew_TA_p<-.5-(1-.5)*t/(1-t)

probharmful<-1-equals(max(max(NBnew,NBnew_TA),0), NBnew)

probharmful_p<-1-equals(max(max(NBnew_p,NBnew_TA_p),0), NBnew_p)

}

**Web appendix 2: WinBUGS code using an inverse Wishart prior for the variance-covariance matrix**

model{

for (i in 1:N){

n[i]<-ncases[i]+ncontrols[i]

ncases[i] ~ dbin(prev[i],n[i])

tp[i] ~ dbin(sens[i],ncases[i])

tn[i] ~ dbin(spec[i],ncontrols[i])

logits[i,1:3] ~ dmnorm(mu[1:3],T[1:3,1:3])

prev[i] <- exp(logits[i,1])/(1+exp(logits[i,1]))

sens[i] <- exp(logits[i,2])/(1+exp(logits[i,2]))

spec[i] <- exp(logits[i,3])/(1+exp(logits[i,3]))

}

#compute summary statistics

pooledprev <- exp(mu[1])/(1+exp(mu[1]))

pooledsens <- exp(mu[2])/(1+exp(mu[2]))

pooledspec <- exp(mu[3])/(1+exp(mu[3]))

tau[1:3,1:3] <- inverse(T[1:3,1:3])

tau2prev<- tau[1,1]

tau2sens<- tau[2,2]

tau2spec<- tau[3,3]

cov1 <- tau[1,2]

corr1 <- cov1/sqrt(tau2sens*tau2prev)

cov2 <- tau[1,3]

corr2 <- cov2/sqrt(tau2spec*tau2prev)

cov3 <- tau[2,3]

corr3 <- cov3/sqrt(tau2spec*tau2sens)

t<-0.2

pooledNB<-pooledsens*pooledprev-(1-pooledspec)*(1-pooledprev)*t/(1-t)

pooledNB_TA<-pooledprev-(1-pooledprev)*t/(1-t)

pooledNB_p<-pooledsens*.5-(1-pooledspec)*(1-.5)*t/(1-t)

pooledNB_TA_p<-.5-(1-.5)*t/(1-t)

\# priors

mu[1:3] ~ dmnorm(mn[1:3],prec[1:3,1:3])

T[1:3,1:3] ~ dwish(R[1:3,1:3],3)

\# Predict new triade of sens and spec and prev

logitsnew[1:3] ~ dmnorm(mu[1:3],T[1:3,1:3])

prevnew<-exp(logitsnew[1])/(1+exp(logitsnew[1]))

sensnew<-exp(logitsnew[2])/(1+exp(logitsnew[2]))

specnew<-exp(logitsnew[3])/(1+exp(logitsnew[3]))

NBnew<-sensnew*prevnew-(1-specnew)*(1-prevnew)*t/(1-t)

NBnew_TA<-prevnew-(1-prevnew)*t/(1-t)

NBnew_p<-sensnew*.5-(1-specnew)*(1-.5)*t/(1-t)

NBnew_TA_p<-.5-(1-.5)*t/(1-t)

probharmful<-1-equals(max(max(NBnew,NBnew_TA),0), NBnew)

probharmful_p<-1-equals(max(max(NBnew_p,NBnew_TA_p),0), NBnew_p)

}
